# Two dimensionless parameters and a mechanical analogue for the HKB model of motor coordination

**DOI:** 10.1007/s00422-021-00879-5

**Published:** 2021-06-05

**Authors:** J. F. Cass, S. J. Hogan

**Affiliations:** Department of Engineering Mathematics, University of Bristol, Bristol, BS8 1UB UK

**Keywords:** Haken–Kelso–Bunz model, Motor coordination, Mechanical analogue, Nondimensionalisation, Bifurcation

## Abstract

The widely cited Haken–Kelso–Bunz (HKB) model of motor coordination is used in an enormous range of applications. In this paper, we show analytically that the weakly damped, weakly coupled HKB model of two oscillators depends on only two dimensionless parameters; the ratio of the linear damping coefficient and the linear coupling coefficient and the ratio of the combined nonlinear damping coefficients and the combined nonlinear coupling coefficients. We illustrate our results with a mechanical analogue. We use our analytic results to predict behaviours in arbitrary parameter regimes and show how this led us to explain and extend recent numerical continuation results of the full HKB model. The key finding is that the HKB model contains a significant amount of behaviour in biologically relevant parameter regimes not yet observed in experiments or numerical simulations. This observation has implications for the development of virtual partner interaction and the human dynamic clamp, and potentially for the HKB model itself.

## Introduction

Coordinated human and animal movement is achieved through complex vascular, skeletal, muscular and neural interactions. The degrees of freedom problem (first recognised by Bernstein (1967)) ask how the high dimensionality of such systems is reduced, enabling an organism to perform macroscopic functional tasks. Coordination dynamics (Fuchs and Jirsa 2007; Kelso 2009) has approached this question through the study of the simplest observable quantity that characterizes coordination: the *relative phase* $\phi$ of two oscillators in periodic motion (Kelso 1995). These oscillators may, for example, represent the fingers or limbs of one or more experimental subjects, teams in group sports or neural oscillators. Stable *in-phase* ($\phi =0$) synchronisation is often found to be the simplest to maintain (Buchanan and Ryu 2006; Kelso 1981), while stable *anti-phase* ($\phi =\pi$) motions are also commonly observed (Bourbousson et al. 2010). Stable *phase-lagged* (intermediate values of $\phi$) states are also found in a variety of situations (Collins and Stewart 1993; Duarte et al. 2012).

In 1985, Haken et al. (1985) proposed a model, which we shall call the *basic* HKB model, for the potential function of the relative phase $\phi$ of two oscillators, which has equilibria at $\phi =0$ and $\phi =\pi$. Variation of the model’s single parameter induces changes in the one-dimensional dynamics derived from the potential function, in particular a loss of stability of the anti-phase mode causing an abrupt shift to the (always stable) in-phase mode. These dynamics have been used to model phase transitions, analogous to the switching of an animal’s gait, originally seen in bimanual finger experiments (Kelso 1981), but also later in interpersonal (Schmidt et al. 1990) and sensorimotor (Kelso et al. 1990) contexts. The suitability of the model for these applications (and its stochastic extension Schöner et al. 1986) has been well established through measurements of critical fluctuations and critical slowing down around the transition (Kelso et al. 1986; Scholz et al. 1987).

The natural next step was to consider the dynamics of the component oscillators. A self-sustaining so-called *hybrid* oscillator (consisting of Rayleigh and Van-der-Pol style damping terms) was found to fit an observed (inverse) monotonic relationship between amplitude and frequency (Kay et al. 1987). This oscillator contains four intrinsic parameters—one linear and two nonlinear damping coefficients and a natural frequency (considered to be a control parameter set by the experimenter, for example a pacing metronome). Many authors have commented on the difficulty in ascribing physical significance to these parameters (Peper et al. 2004). When coupled nonlinearly, two hybrid oscillators were shown (Haken et al. 1985) to produce the relative phase dynamics of the potential in the basic HKB model (under slowly varying amplitude and rotating wave approximations, and considering equal and constant amplitudes). The coupling contains additional parameters multiplying its linear and nonlinear terms, whose physical significance has again been difficult to determine. We call this model the *full* HKB model.

To make analytical progress, an *approximate* HKB (aHKB) model was obtained by averaging the system of coupled oscillators (Leise and Cohen 2007), under the assumption of weakly nonlinear damping and weakly nonlinear coupling.^1^ Such analysis can then be used to verify any numerical work and to shed light on the processes that give rise to the observed behaviour.

A bifurcation analysis of the full HKB model has been carried out (Avitabile et al. 2016). This approach considers arbitrary coupling strengths, together with no constraints on any other parameter. In this way, the full range of qualitative behaviour in the model can be explored, as well as uncovering the transitions (bifurcations) between such behaviours as parameters are varied. This is part of the general approach to studying such systems, known as nonlinear dynamics (Strogatz 2018, which has had an extensive and prolonged impact in many fields. Such an approach has already been used to create a dynamical framework for motor behaviour, using functional architectures and structured flows on manifolds (Huys et al. 2014).

As well as describing and predicting the dynamics of social coordination between two people (Kay et al. 1987), the HKB model has also been used to study real-time interaction between one human and a machine, via virtual partner interaction (VPI) (Kelso et al. 2009), where novel behaviours were found, and its extension to the human dynamic clamp (HDC) (Dumas et al. 2014). Multiple human players modelled as networks of HKB oscillators have also been studied (Alderisio et al. 2016).

Hence, for VPI and HDC to be effective, or to aid in understanding the more complex situation of many coupled oscillators, it is important that all the different types of behaviour contained within the two-oscillator HKB model are explored and catalogued and the overall dynamics understood. In particular, it is important to understand the parameter regimes where the approximate HKB model is valid and how results differ when the full HKB model is considered.

In this paper, we extend work on the approximate HKB model (Leise and Cohen 2007) and compare it to our own bifurcation analysis of the full HKB system. We find that the dynamics of the approximate HKB model are governed by just two dimensionless parameters: $\mu$ (17) the ratio of the linear damping to linear coupling and $\kappa$ (18) the ratio of nonlinear damping to nonlinear coupling. This nondimensionalisation clarifies the weakly nonlinear regimes where we see the emergence of two new synchronisation behaviours; bistability of in-phase, anti-phase and phase-lagged solutions, without the need for extensive computations. We find excellent agreement between these analytical solutions and numerical computations (for moderate amplitudes), and through this process uncover extra solution branches not present in the analysis of Avitabile et al. (2016), leading us to provide, for the first time, the complete picture of the dynamics in the parameter range chosen by these authors.

The new results in this paper are:

- the discovery of normal modes (5) in the linear HKB model (4) and the mechanical analogy to which it corresponds (Fig. 2),
- the nondimensionalisation (15) that reduces the aHKB model (13) to an Eq. (16) with just two dimensionless parameters,
- determination of the existence and stability of steady states of this nondimensional aHKB model (16) in terms of arbitrary parameter values,
- the use of these steady states (Fig. 4) to predict the dynamics in the full HKB model in arbitrary parameter ranges,
- an alternative nondimensionalisation more suited to comparison with numerical computations (Sect. 4.1),
- our own numerical computations that provide the full picture (Sect. 4) of the dynamics in the parameter ranges used in Avitabile et al. (2016),
- a mechanical analogue (Fig. 5) of the nondimensional aHKB model (16),
- the reduction in a very general form of aHKB model (88) to one with just two dimensionless parameters.

Our paper is organised as follows. In Sect. 2, we present the full HKB model (Haken et al. 1985). Then, we introduce the linear HKB model and a corresponding mechanical analogue, which does not appear to have been considered before. This section also contains a nondimensionalisation that reduces the aHKB model to a system with just two dimensionless parameters.

In Sect. 3, we determine general existence and stability criteria of the steady states of the nondimensionalised aHKB model in terms of $\kappa$ and $\mu$ that correspond to synchronisation. In Sect. 4, we use these results to explain and extend recent numerical continuation results (Avitabile et al. 2016) of the full HKB model. This section contains Fig. 4, possibly the most important figure in the paper. It can be used to predict the behaviour of the weakly coupled, weakly damping aHKB system in an arbitrary range of parameters. Much of this behaviour has not yet been seen in experiments or numerical simulations to date. In Sect. 5, we discuss our results and present our conclusions. The paper ends with a number of appendices, including Appendix E where we show that a large family of weakly damped, weakly coupled HKB oscillators can be reduced to our nondimensional aHKB system, suggesting that its dynamics are more universal than the particular form of HKB model that is the current paradigm.

## The Haken–Kelso–Bunz model

In its most general form, the Haken–Kelso–Bunz (HKB) model (Haken et al. 1985) of two coupled nonlinear oscillators is given by the ordinary differential equations

1 $$\begin{aligned} \ddot{x}_1 + \omega ^2 x_1&= h(x_1,{\dot{x}}_1) + J(x_1-x_2,{\dot{x}}_1-{\dot{x}}_2), \nonumber \\ \ddot{x}_2 + \omega ^2 x_2&= h(x_2,{\dot{x}}_2) + J(x_2-x_1,{\dot{x}}_2-{\dot{x}}_1). \end{aligned}$$

where differentiation with respect to time is denoted by a dot, $x_1, x_2$ are the oscillator amplitudes, frequency $\omega >0$ (the control or pacing parameter), $h(x,{\dot{x}})$ is a nonlinear damping term and $J(x_1-x_2,{\dot{x}}_1-{\dot{x}}_2)$ is a nonlinear coupling term.

Much work has been done in establishing the correct form of $h(x,{\dot{x}})$ and $J(x_1-x_2,{\dot{x}}_1-{\dot{x}}_2)$ to use when seeking to explain human-human or human-virtual player interactions. The following form covers all the different types of full HKB model (Haken et al. 1985) studied in the subsequent literature:

2 $$\begin{aligned} \ddot{x}_1 + \omega ^2 x_1&= (\gamma - \alpha x_1^2 - \beta {\dot{x}}_1^2) {\dot{x}}_1 \nonumber \\&\quad + [a + b(x_1-x_2)^2 \nonumber \\&\quad +c({\dot{x}}_1-{\dot{x}}_2)^2 ]({\dot{x}}_1 - {\dot{x}}_2), \nonumber \\ \ddot{x}_2 + \omega ^2 x_2&= (\gamma - \alpha x_2^2 - \beta {\dot{x}}_2^2) {\dot{x}}_2 \nonumber \\&\quad + [a + b(x_2-x_1)^2 \nonumber \\&\quad +c({\dot{x}}_1-{\dot{x}}_2)^2 ]({\dot{x}}_2 - {\dot{x}}_1), \end{aligned}$$

where $\gamma$ is a linear damping coefficient, $\alpha$ is the Van der Pol damping coefficient, $\beta$ is the Rayleigh damping coefficient, and *a* is a linear coupling coefficient, *b* and *c* are nonlinear coupling coefficients. The dimensions of these coefficients are given by

3 $$\begin{aligned}{}[\gamma ] = T^{-1}, \, [\alpha ] = L^{-2} T^{-1}, \, [\beta ]= L^{-2}T, \nonumber \\ [a] = T^{-1}, \, [b] = L^{-2}T^{-1},\,[c]=L^{-2}T. \end{aligned}$$

The rationale for the form of (1) and (2) is fully explained in Haken et al. (1985), to which the interested reader is referred. Terms in these equations are needed to describe oscillations; these are given by the left hand side of both (1) and (2). Then, since *“movement has a more or less stable amplitude, the equations must be nonlinear”* (Haken et al. 1985, p. 351) and that experimentally this amplitude *“drops* $\cdots$ *with increasing* $\omega$” (Haken et al. 1985, p. 352). One form of $h(x,{\dot{x}})$ that meets these criteria is given by $h(x,{\dot{x}})=(\gamma - \alpha x^2 - \beta {\dot{x}}^2) {\dot{x}}$. The coupling term *J* was a subject of great discussion in Haken et al. (1985), with the form chosen *“in the sense of a minimal model”* (Haken et al. 1985, p. 353) that produced *“the correct phase relationship between the two oscillators”* (Haken et al. 1985, p. 352).

In (2), the nonlinear damping term $h(x,{\dot{x}})$ is *softening* for positive parameter values $\alpha , \, \beta$, whereas the nonlinear coupling term $J(x_1-x_2,{\dot{x}}_1-{\dot{x}}_2)$ is *hardening* for positive parameter values $b, \, c$. The values of $\gamma , \, \alpha ,\, \beta , \, a,\, b,\, c$ are either chosen by fitting with experimental data or selected in parameter sweeps. In Table 1, we give representative values of the other parameters selected from the literature. All authors except (Leise and Cohen 2007) set $c=0$. In most papers, the pacing frequency $\omega \in [1,10]$, although values as high as $\omega =12\pi$ have been used. Theoretical analysis is simplified under the assumption $\gamma \ll \omega$ (Haken et al. 1985).

**Table 1:** Representative values of parameters $\gamma ,\, \alpha ,\, \beta ,\, a, \, b$ used in the full HKB model (2) selected from the literature

References	$\gamma$	$\alpha$	$\beta$	*a*	*b*
Avitabile et al. (2016)	$\in [-2,8]$	1	1	$\pm 0.5$	$\pm 0.5$
Fink et al. (2000)	1	1	1	−0.2	0.5
Haken et al. (1985)	1	0	1	−0.2	0.2
Jirsa et al. (2000)	1	1	1	−0.2	0.2
Leise and Cohen (2007)	0.5	0.38	0.001	−0.05	0.036
Słowiński et al. (2016)	0.641	12.457	0.008	$\in [-15,15]$	1
Varlet et al. (2012)	0.7	1	1	−3.2	3.2

All authors except (Leise and Cohen 2007) set $c=0$

**Fig. 1:**
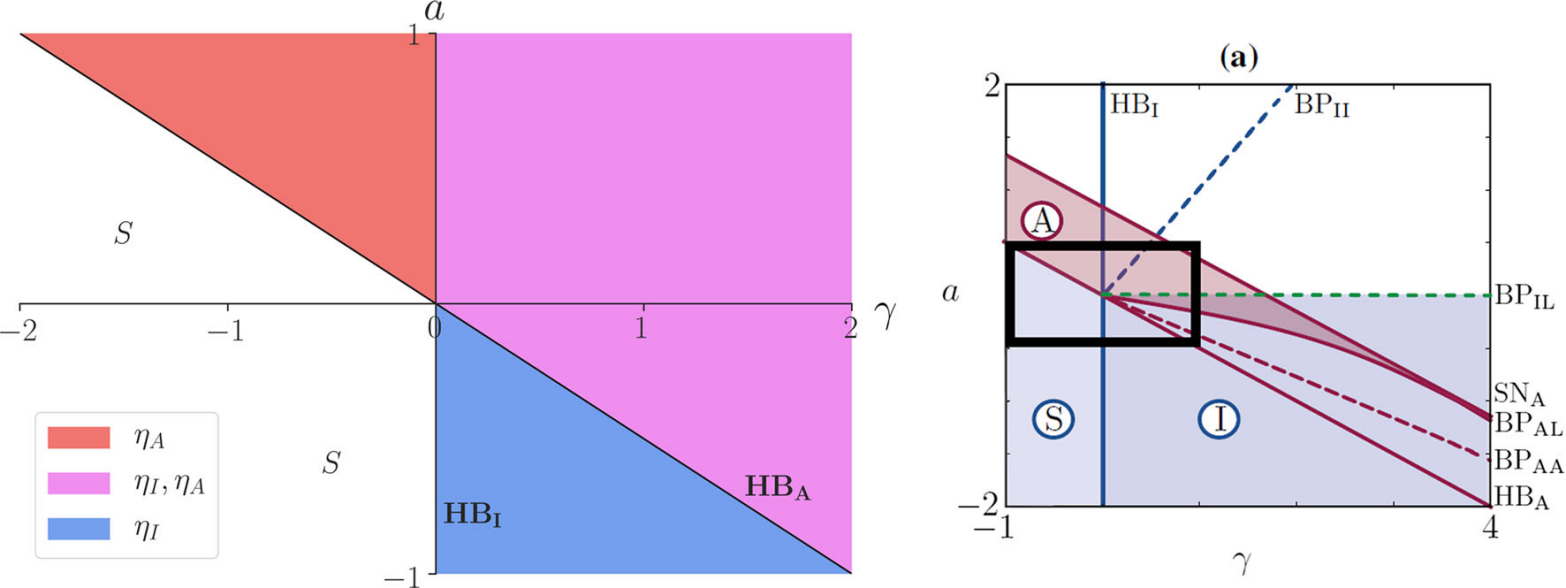
(Left pane) Regions of $(\gamma ,a)$ parameter space in which in-phase $\eta _I$ and anti-phase $\eta _A$ normal modes are *unstable*, where we find in-phase and anti-phase synchronisation. In region *S*, we find stable steady states only and hence no limit cycles. We find that $HB_I: \gamma =0$ and $HB_A: 2a +\gamma = 0$, both independent of the nonlinear coefficients. (Right pane) Numerical continuation results from (Avitabile et al. 2016, Figure 5a), with nonlinear coupling term $b=0.5$. The lines $BP_{II}$, $BP_{IL}$, $SN_A$, $BP_{AL}$ and $BP_{AA}$ in this pane are nonlinear effects. Most studies of the full HKB model (2) are carried out in the fourth quadrant of these figures (see Table 1)

### The linear HKB model

We begin by studying the *linear* HKB model, obtained by neglecting the nonlinear terms in (2) to get

4 $$\begin{aligned} \ddot{x}_1 + \omega ^2 x_1&= \gamma {\dot{x}}_1 + a ({\dot{x}}_1 - {\dot{x}}_2), \nonumber \\ \ddot{x}_2 + \omega ^2 x_2&= \gamma {\dot{x}}_2 + a({\dot{x}}_2 - {\dot{x}}_1). \end{aligned}$$

This model does not seem to have been considered before, within the context of the HKB system. But it sheds important light on both the origin of in-phase and anti-phase synchronisation and the nature of the coupling.

The linear HKB model (4) consists of two normal modes, given by $\eta _I \equiv x_1 + x_2$ and $\eta _A \equiv x_1 - x_2$, which satisfy the equations

5 $$\begin{aligned} \ddot{\eta }_I - \gamma {\dot{\eta }}_I + \omega ^2 \eta _I&= 0 ,\nonumber \\ \ddot{\eta }_A - (2a +\gamma ) {\dot{\eta }}_A + \omega ^2 \eta _A&= 0, \end{aligned}$$

corresponding to in-phase and anti-phase motion, respectively. The resulting instability chart in $(\gamma , a)$ parameter space is shown in Fig. 1 (left pane), with the analytic details given in Appendix A. Our analytic results should be compared with (Avitabile et al. 2016, Fig. 5a), reproduced here in Fig. 1 (right pane) which was obtained by fixing the value of the nonlinear coupling at $b=0.5$ and performing a numerical continuation in $(\gamma ,a)$.

These two panes show agreement in the lines $HB_I$ and $HB_A$ (where unstable modes become saturated limit cycles). We have shown that these lines, which are a fundamental part of the model originating in the linear HKB equations (4), do not change as the nonlinear coefficients $\alpha$, $\beta$, *b*, *c* vary.

The lines $BP_{II}$, $BP_{IL}$, $SN_A$, $BP_{AL}$ and $BP_{AA}$ in the right pane are all nonlinear effects, which do vary as the nonlinear coefficients vary and so are absent from the left pane. In Sect. 3 below, we will obtain analytic expressions for the weakly nonlinear versions of these lines (except $SN_A$ which occurs at very large amplitudes only, outside the range of validity of that analysis).

### Mechanical analogue of the linear HKB model

**Fig. 2:**
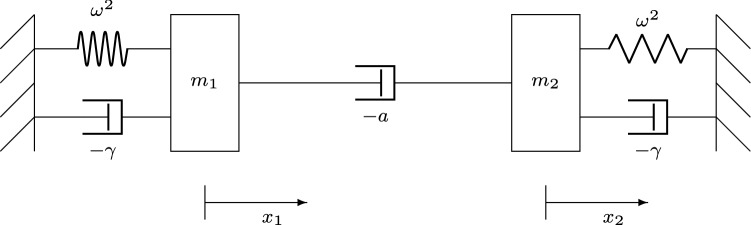
Mechanical analogue of the linear HKB model (4). Both masses $m_{1,2}$ are connected to rigid surfaces by a linear spring, with spring constant $\omega ^2$, and a linear dashpot, with damping coefficient $-\gamma$. The masses themselves are connected by another linear dashpot, with linear damping coefficient $-a$. The governing equations of this system are precisely the same as those of the linear HKB model (4), when $m_1=m_2=1$

The form of (4) leads us to propose a mechanical analogue of the linear HKB model, shown in Fig. 2.

Mass $m_1$ is connected to a rigid surface by a linear spring, with spring constant $\omega ^2$, and a linear dashpot, with damping coefficient^2^$-\gamma$. Another mass $m_2$ is connected in an identical way to a separate rigid surface. The two masses are themselves connected by a linear dashpot whose damping coefficient is $-a$. When $m_1=m_2=1$, the governing equations of the system in Fig. 2 are *precisely* those of the linear HKB model (4).

In this analogy, the coupling term $a ({\dot{x}}_1 - {\dot{x}}_2)$ can be seen to be a form of damping. Clearly, when both $a, \, \gamma >0$, there is negative damping in the whole system and the equilibrium $x_1=x_2=0$ is impossible. Similarly, if both $a, \, \gamma <0$, we have positive damping, and all oscillations die out as energy is taken out of the system. We see both these cases in Fig. 1 (in the first and third quadrants, respectively).

It is clear why the in-phase motion is independent of the coupling coefficient *a* and dependent on the sign of $\gamma$. As $x_{1,2}$ move in phase, the coupling has no influence and the only damping that can affect the motion is $\gamma$.

On the other hand, anti-phase motion is clearly dependent on the relative size of the coefficients $a, \, \gamma$. As we shall see in Sect. 2.4, the ratio of these two coefficients plays a crucial role in the dynamics of the full HKB model (2).

### Hopf bifurcations

We now consider how in-phase and anti-phase motions manifest themselves in the full (nonlinear) HKB model (2). For in-phase motion, we set $x_1=x_2=x_I$ in (2) to find

6 $$\begin{aligned} \ddot{x}_I + \omega ^2 x_I&= (\gamma - \alpha x^2_I - \beta {\dot{x}}^2_I) {\dot{x}}_I. \end{aligned}$$

Under the assumption of weak nonlinearity, it can be shown (Haken et al. 1985; Leise and Cohen 2007) that a Hopf bifurcation $HB_I$ occurs in (6) at $\gamma =0$, to produce equal amplitude in-phase synchronisation with amplitude $x_I=r_I$ given by

7 $$\begin{aligned} r_I&= 2\sqrt{\frac{\gamma }{\alpha +3\beta \omega ^2}}, \end{aligned}$$

provided $\frac{\gamma }{\alpha +3\beta \omega ^2}>0$. $HB_I$ is supercritical, degenerate, subcritical (Avitabile et al. 2016) for $\alpha +3\beta \omega ^2 >0, \, =0, \, <0$, respectively.

For anti-phase motion, we set $x_1=-x_2=x_A$ in (2) to find

8 $$\begin{aligned}&\ddot{x}_A + \omega ^2 x_A = [(\gamma + 2a) - (\alpha -8b)x^2_A \nonumber \\&\quad - (\beta -8c){\dot{x}}^2_A] {\dot{x}}_A. \end{aligned}$$

So, under the assumption of weak nonlinearity, by relabelling terms in (6), it can be seen that a Hopf bifurcation $HB_A$ occurs in (8) at $\gamma +2a=0$, to produce equal amplitude anti-phase synchronisation with amplitude $x_A=r_A$ given by

9 $$\begin{aligned} r_A&= 2\sqrt{\frac{\gamma +2a}{(\alpha +3\beta \omega ^2)-8(b+3c\omega ^2)}}, \end{aligned}$$

provided $\frac{\gamma +2a}{(\alpha +3\beta \omega ^2)-8(b+3c\omega ^2)}>0$. Hence, $HB_A$ must be supercritical, degenerate, subcritical for $(\alpha +3\beta \omega ^2)-8(b+3c\omega ^2) >0, \, =0, \, <0$, respectively. Both $HB_I: \gamma =0$ and $HB_A: \gamma +2a=0$ are shown in Fig. 1.

### The nondimensional approximate HKB (aHKB) model

In this section, we present the approximate HKB (aHKB) model, as derived in Haken et al. (1985) and show that it can be represented by a set of equations involving only two dimensionless parameters.

Let us rewrite (2) in terms of a small parameter $\epsilon \ll 1$ and suitably redefined damping and coupling coefficients:

10 $$\begin{aligned} \ddot{x}_1 + \omega ^2 x_1&= \epsilon [(\gamma - \alpha x_1^2 - \beta {\dot{x}}_1^2) {\dot{x}}_1 \nonumber \\&\quad + [a + b(x_1-x_2)^2 \nonumber \\&\quad + c({\dot{x}}_1-{\dot{x}}_2)^2 ]({\dot{x}}_1 - {\dot{x}}_2)], \nonumber \\ \ddot{x}_2 + \omega ^2 x_2&= \epsilon [(\gamma - \alpha x_2^2 - \beta {\dot{x}}_2^2) {\dot{x}}_2 \nonumber \\&\quad + [a + b(x_2-x_1)^2 \nonumber \\&\quad + c({\dot{x}}_2-{\dot{x}}_1)^2 ]({\dot{x}}_2 - {\dot{x}}_1)]. \end{aligned}$$

We look for solutions of the form

11 $$\begin{aligned} x_1(t, \epsilon )&= x_1^0(\tau , T) + \epsilon x^1_1(\tau , T) + O(\epsilon ^2) \nonumber \\ x_2(t, \epsilon )&= x_2^0(\tau , T) + \epsilon x_2^1(\tau , T) + O(\epsilon ^2), \end{aligned}$$

where $\tau =t$ and $T = \epsilon t$ represent two time scales, and $x_{1,2}^i= O(1), \, (i=0,1)$. Then, we take

12 $$\begin{aligned} x_i^0(\tau ,T)&= r_i(T) \cos (\omega \tau + \phi _i(T)), \, (i=1,2), \end{aligned}$$

corresponding to a limit cycle of slowly varying amplitude *r*(*T*) and phase $\phi (T)$.

Using averaging (Leise and Cohen 2007, eq. (8)–(10)) or two-timing (Cass 2019), we obtain the *approximate* HKB (aHKB) model:

13 $$\begin{aligned} 8 {\dot{r}}_1&= [4 \gamma - (\alpha +3\beta \omega ^2) r_1^2]r_1 \nonumber \\&\quad + [4 a + (b+3c\omega ^2)(r_1^2 + r_2^2 \nonumber \\&\quad - 2 r_1 r_2 \cos \phi )](r_1 - r_2 \cos \phi ), \nonumber \\ 8 {\dot{r}}_2&= [4 \gamma - (\alpha +3\beta \omega ^2) r_2^2]r_2 \nonumber \\&\quad + [4 a + (b+3c\omega ^2)(r_1^2 + r_2^2 \nonumber \\&\quad - 2 r_1 r_2 \cos \phi )](r_2 - r_1 \cos \phi ), \nonumber \\ 8r_1r_2{\dot{\phi }}&= (r_1^2 + r_2^2)\sin \phi [4a \nonumber \\&\quad + (b+3c\omega ^2)(r_1^2 + r_2^2 - 2 r_1 r_2 \cos \phi )], \end{aligned}$$

where

14 $$\begin{aligned} \phi = \phi _1-\phi _2 \end{aligned}$$

is the relative phase between the two oscillators.

The aHKB model (13) depends on six parameters: $a,b,c,\alpha ,\beta ,\gamma$. We have found a scaling that greatly simplifies (13), leading to a model with just two *dimensionless* parameters.

Assume, in line with experimental data, that each of $\gamma , \, \alpha , \, \beta$ is positive (see Table 1). One solution of (13) is given (Leise and Cohen 2007) by $r_1=r_2=r_I, \, \phi =0$, where $r_I$ is given by (7), corresponding to equal amplitude in-phase synchronisation.

Let us introduce nondimensionalised amplitudes $R_1, \, R_2$ and time *s* as follows:

15 $$\begin{aligned} R_i = \frac{r_i}{r_I}, \, (i=1,2); \quad s=\frac{1}{2}\gamma t. \end{aligned}$$

Then, equations (13) become

16 $$\begin{aligned} \dot{R_1}&= R_1 - R_1^3 + (R_1 - R_2 \cos \phi )(\mu \nonumber \\&\quad + \kappa (R_1^2 + R_2^2 - 2 R_1 R_2 \cos \phi )), \nonumber \\ \dot{R_2}&= R_2 - R_2^3 + (R_2 - R_1 \cos \phi )(\mu \nonumber \\&\quad + \kappa (R_1^2 + R_2^2 - 2 R_1 R_2 \cos \phi )), \nonumber \\ R_1R_2 {\dot{\phi }}&= (R_1^2 + R_2^2)\sin \phi (\mu \nonumber \\&\quad + \kappa (R_1^2 + R_2^2 - 2 R_1 R_2 \cos \phi )), \end{aligned}$$

where differentiation with respect to *s* is (still) denoted by a dot and the dimensionless parameters $\mu , \, \kappa$ are given by

17 $$\begin{aligned} \mu \equiv \frac{a}{\gamma }, \end{aligned}$$

18 $$\begin{aligned} \kappa \equiv \frac{b+3c\omega ^2}{\alpha +3\beta \omega ^2} = \frac{d}{\delta }. \end{aligned}$$

We see that $\mu$ is the ratio of the *linear* damping coefficient $\gamma$ to the *linear* coupling coefficient *a*. We have already seen the importance of $\mu$ in the mechanical analogue (Fig. 2) of the linear HKB model (4). We can think of $\kappa$ as being the ratio of the *combined nonlinear* coupling coefficient *d*, where

19 $$\begin{aligned} d \equiv \frac{1}{4}(b+3c\omega ^2), \end{aligned}$$

to the *combined nonlinear* damping coefficient $\delta$, where^3^

20 $$\begin{aligned} \delta \equiv \frac{1}{4}(\alpha +3\beta \omega ^2). \end{aligned}$$

To the best of our knowledge, the derivation of (15)–(18) has not been reported before in the literature.^4^

In this section, we have considered the linear HKB model (4). We have shown the presence of normal modes (5), how their loss of stability corresponds to the generation of stable limit cycles and the central role they play in understanding the stability structure of the full HKB model (Fig. 1). We have also shown that the linear HKB model has a mechanical analogue, Fig. 2, which sheds light on the fundamental mechanisms behind the HKB model. We conclude this section with a note of the importance of dimensionless parameters in the HKB model. This significant development means (for example) that we will get the same results for $a=a_0, \gamma =\gamma _0$ as we would for $a=ka_0, \gamma =k\gamma _0$ for *any* value of $k \ne 0$. Hence, we can search parameter space far more efficiently to find dynamics relevant to the application of the HKB model under consideration.

## Steady states of the nondimensional aHKB model

To understand the dynamics of (16) as dimensionless parameters $\mu , \, \kappa$ vary, we consider the existence and stability of steady states of these equations. Stable steady states of the dimensionless aHKB model correspond to stable limit cycles of the full HKB model. We revisit and adapt the approach in Leise and Cohen (2007) to obtain results in an arbitrary range of parameters. We will see in the following analysis the emergence of two new synchronisation behaviours not possible in the linear HKB model; bistability of in-phase and anti-phase, and phase-lagged synchronisation.

### Existence of steady states

Let us denote steady states of (16) by

$$\begin{aligned} (R_1,R_2,\phi )=(R_1^*, R_2^*, \phi ^*), \end{aligned}$$

corresponding to limit cycles of constant, possibly different, amplitudes $R_1^*, R_2^*$ separated by a constant phase difference $\phi ^*$. Since therefore ${{\dot{\phi }}}=0$, we must have from (16) either

21 $$\begin{aligned} \sin \phi&= 0 \end{aligned}$$

or

22 $$\begin{aligned} \mu + \kappa (R_1^2 + R_2^2 - 2 R_1 R_2\cos \phi )&= 0. \end{aligned}$$

If we assume (21) holds, then $\phi ^*=0, \pi$. Let $\phi ^*=0$, corresponding to in-phase motion. From (16), we must have

23 $$\begin{aligned} 0&= R_1 - R_1^3 \nonumber \\&\quad + (R_1 - R_2)(\mu + \kappa (R_1^2 + R_2^2 - 2 R_1 R_2)) \end{aligned}$$

24 $$\begin{aligned} 0&= R_2 - R_2^3 \nonumber \\&\quad + (R_2 - R_1)(\mu + \kappa (R_1^2 + R_2^2 - 2 R_1 R_2)) \end{aligned}$$

We solve these equations to find three possibilities for $(R_1^*, R_2^*, \phi ^*)$ given by:

25 $$\begin{aligned} I&:= (1, 1, 0), \end{aligned}$$

26 $$\begin{aligned} N_0^\pm&:= \biggl (\frac{1}{2}\sqrt{\frac{1 + 3 \mu + 4 \kappa }{1+\kappa }} \pm \frac{1}{2}\sqrt{\frac{1-\mu }{1+\kappa }}, \nonumber \\&\qquad \qquad \frac{1}{2}\sqrt{\frac{1 + 3 \mu + 4 \kappa }{1+\kappa }} {\mp } \frac{1}{2}\sqrt{\frac{1-\mu }{1+\kappa }}, 0\biggr ), \end{aligned}$$

27 $$\begin{aligned} Z_0&:= (0,0,0). \end{aligned}$$

Steady state *I* has in-phase equal amplitudes, exists $\forall \, \mu , \kappa$ and corresponds to $r_I$ in (9). Steady states $N_0^\pm$ have in-phase *unequal* amplitudes and steady state $Z_0$ is degenerate.

Still assuming (21), but now with $\phi ^*=\pi$, this case corresponds to anti-phase motion. Again there are three possibilities for $(R_1^*, R_2^*, \phi ^*)$ given by :

28 $$\begin{aligned} A&:= \left( \sqrt{\frac{1+2\mu }{1-8\kappa }},\sqrt{\frac{1+2\mu }{1-8\kappa }}, \pi \right) , \end{aligned}$$

29 $$\begin{aligned} N_\pi ^\pm&:= \biggl (\frac{1}{2}\sqrt{\frac{1-\mu }{1+\kappa }} \pm \frac{1}{2}\sqrt{\frac{1 + 3 \mu + 4 \kappa }{1+\kappa }},\nonumber \\&\qquad \qquad \frac{1}{2}\sqrt{\frac{1-\mu }{1+\kappa }} {\mp } \frac{1}{2}\sqrt{\frac{1 + 3 \mu + 4 \kappa }{1+\kappa }}, \pi \biggr ), \end{aligned}$$

30 $$\begin{aligned} Z_\pi&:= (0,0,\pi ). \end{aligned}$$

Steady state *A* has anti-phase equal amplitudes. Steady states $N_\pi ^\pm$ have anti-phase *unequal* amplitudes, and steady state $Z_\pi$ is degenerate.

Finally, if (22) holds, there are three possibilities for $(R_1^*, R_2^*, \phi ^*)$ corresponding to *phase-lagged* steady states given by:

31 $$\begin{aligned} L^\pm&:= \left( 1, 1, \pm \cos ^{-1}\left[ 1+ \frac{\mu }{2\kappa }\right] \right) , \end{aligned}$$

32 $$\begin{aligned} N_{\pm \frac{\pi }{2}}^1&:= \left( 1, 0, \pm \frac{\pi }{2}\right) , \quad N_{\pm \frac{ \pi }{2}}^2 := \left( 0, 1, \pm \frac{\pi }{2}\right) , \end{aligned}$$

33 $$\begin{aligned} Z_\phi&:= (0,0,\phi ). \end{aligned}$$

Both of $L^\pm$ have equal oscillation amplitudes, whereas the $N_{\pm \frac{\pi }{2}}^{(1,2)}$ have *unequal* oscillation amplitudes. Steady state $Z_\phi$ is degenerate.

Table 2 summarises the steady states of (16), together with those regions of $(\kappa , \mu )$ parameter space in which they exist.

**Table 2:** Steady states of (16), corresponding to limit cycles of (2), grouped according to the relative size of $R_1^*,R_2^*$

	**Name**	$\mathbf {R_1^*}$	$\mathbf {R_2^*}$	$\varvec{\phi ^*}$	**Existence regions**
$$\mathbf {R_1^*=R_2^* \ne 0}$$					
1	*I*	1	1	0	Arbitrary $\kappa , \, \mu$
2	*A*	$\sqrt{\frac{1 + 2 \mu }{1 - 8 \kappa }}$	$\sqrt{\frac{1 + 2 \mu }{1 - 8 \kappa }}$	$\pi$	$\kappa < \frac{1}{8}$, $\mu > - \frac{1}{2}$ or $\kappa > \frac{1}{8}$, $\mu < - \frac{1}{2}$
3	$L^\pm$	1	1	$\pm \cos ^{-1}\left( 1 + \frac{\mu }{2 \kappa }\right)$	$0< \mu < -4 \kappa$ or $-4 \kappa< \mu < 0$
$$\mathbf {R_1^*\ne R_2^* (\ne 0)}$$					
4	$N_0^\pm$	${\frac{1}{2}\sqrt{\frac{1 + 3 \mu + 4 \kappa }{1+\kappa }} \pm \frac{1}{2}\sqrt{\frac{1-\mu }{1+\kappa }}}$	${\frac{1}{2}\sqrt{\frac{1 + 3 \mu + 4 \kappa }{1+\kappa }} {\mp } \frac{1}{2}\sqrt{\frac{1-\mu }{1+\kappa }}}$	0	$\kappa< -1, \, \mu > 1, \,\, \mu + \kappa < 0$ or $\kappa> -1, \,\, \mu < 1, \,\, \mu + \kappa > 0$
5	$N_\pi ^\pm$	${\frac{1}{2}\sqrt{\frac{1-\mu }{1+\kappa }} \pm \frac{1}{2}\sqrt{\frac{1 + 3 \mu + 4 \kappa }{1+\kappa }}}$	${\frac{1}{2}\sqrt{\frac{1-\mu }{1+\kappa }} {\mp } \frac{1}{2}\sqrt{\frac{1 + 3 \mu + 4 \kappa }{1+\kappa }}}$	$\pi$	$\kappa< -1, \,\, 3\mu + 4\kappa + 1 <0 , \,\, \mu + \kappa > 0$ or $\kappa> -1, \,\, 3\mu + 4\kappa + 1 >0, \,\, \mu + \kappa < 0$
6	$N_{\pm \frac{\pi }{2}}^{1,2}$	1/0	0/1	$\pm \frac{\pi }{2}$	$\mu + \kappa = 0$
$$\mathbf {R_1^*=R_2^*=0}$$					
7	$Z_0$	0	0	0	Arbitrary $\kappa , \,\, \mu$
8	$Z_\pi$	0	0	$\pi$	Arbitrary $\kappa , \,\, \mu$
9	$Z_\phi$	0	0	Arbitrary	$\mu =0$

Rows 1–5 correspond to (Leise and Cohen 2007, Table 1, rows 2–6); rows 7–9 cover (Leise and Cohen 2007, Table 1, row 1). But (Leise and Cohen 2007, Table 1) has no equivalent of row 6 and (Leise and Cohen 2007, Table 1, row 7) is not a solution of (16)

A similar table was given in (Leise and Cohen (2007), Table 1). However, there are some differences^5^ and our nondimensionalisation was not carried out.

### Stability of steady states

**Fig. 3:**
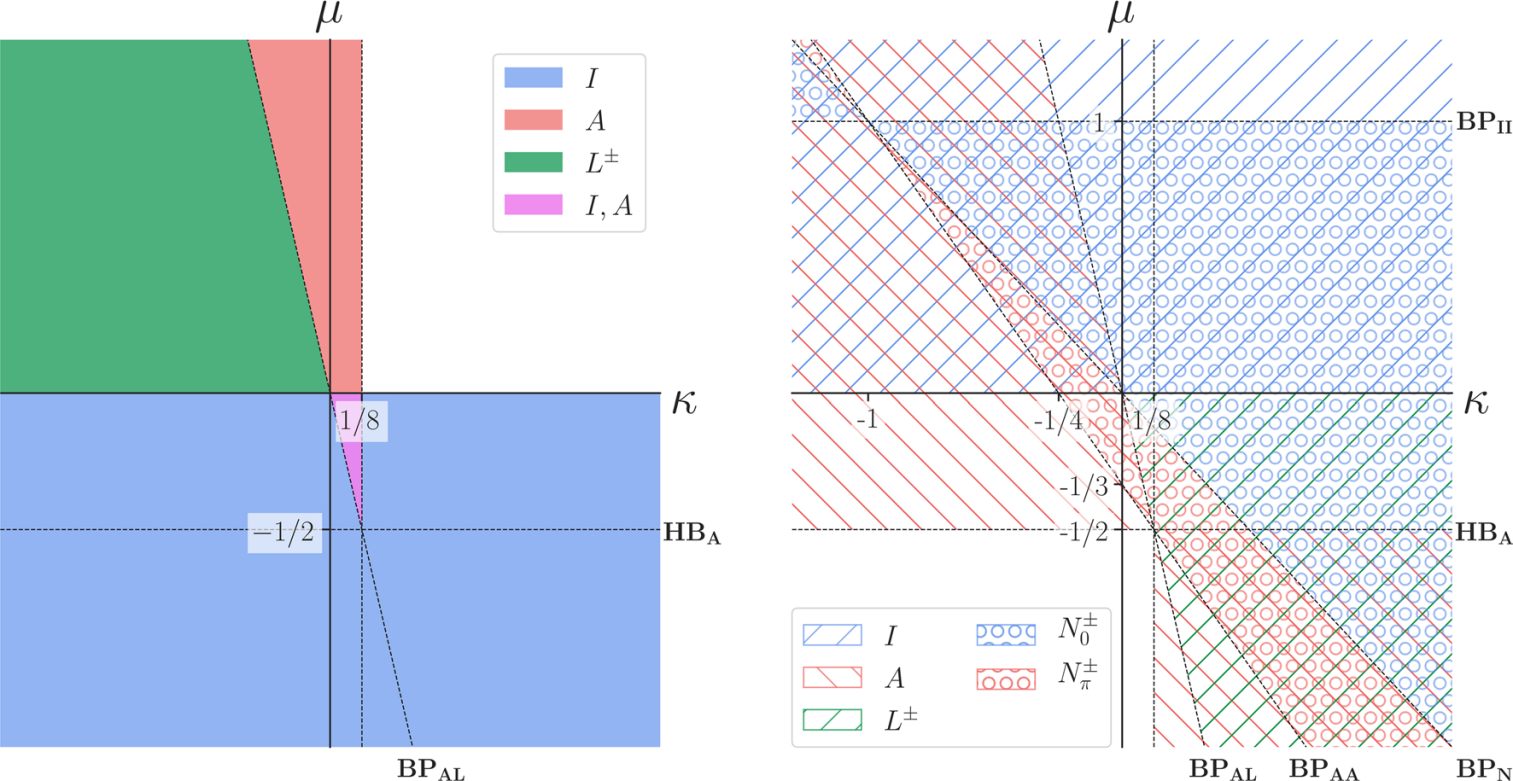
Stability regions (on the left) and instability regions (on the right) of steady states $I, \, A, \, L^\pm , \, N_0^\pm , \, N_{\pi }^\pm$ of (16), where we assume that $\gamma , \alpha , \beta >0$. The labelled lines correspond to $HB_A: \mu =-\frac{1}{2}$, $BP_{AL}: \mu +4\kappa =0$, $BP_{II}: \mu =1$, $BP_{AA}: 3\mu +4\kappa +1=0$, $BP_N: \mu +\kappa =0$. Stable *I* and *A* co-exist and are stable in the fourth quadrant on the left in the purple region between the lines $\mu =0, \, \kappa = \frac{1}{8}, \, \mu +4\kappa =0$

In this section, we consider the stability properties in the $(\kappa ,\mu )$ plane of the steady states of (16) found in the previous section. The regions of stability/instability we identify in this section are given in Fig. 3. As before, we adapt the approach of Leise and Cohen (2007).

First, we consider the three cases of equal amplitude synchronisation $I, \, A, \, L^{\pm }$ (the first three rows of Table 2) given by (25), (28) and (31), respectively.

For *I*, the case of equal amplitude in-phase synchronisation, the eigenvalues $\lambda _i, \, (i=1,2,3)$ are given by Cass (2019)

34 $$\begin{aligned} \lambda _1 = -2, \; \lambda _2 = 2(\mu -1), \; \lambda _3 = 2\mu . \end{aligned}$$

Hence, *I* is stable for $\mu <0$ and unstable for $\mu >0$.

For *A*, the case of equal amplitude anti-phase synchronisation, the eigenvalues are given by Cass (2019)

35 $$\begin{aligned} \lambda _1&= -2(1 + 2\mu ), \; \lambda _2 = 2 \left( \frac{1+3\mu +4\kappa }{8\kappa -1}\right) , \nonumber \\ \lambda _3&= 2 \left( \frac{\mu + 4\kappa }{8\kappa - 1}\right) . \end{aligned}$$

Hence, *A* is stable when $\mu > -\frac{1}{2}$ between the lines $\kappa =\frac{1}{8}$ and $\mu +4\kappa =0$ and unstable either when $\kappa >\frac{1}{8}$, $\mu <-\frac{1}{2}$ or when $\kappa <\frac{1}{8}$ between the lines $\mu =-\frac{1}{2}$ and $\mu +4\kappa =0$. From (17), we see that $\mu = -\frac{1}{2}$ corresponds to the line $HB_A$ in Fig. 1.

For $L^\pm$, the case of equal amplitude phase-lagged synchronisation, the eigenvalues are given by Cass (2019)

36 $$\begin{aligned} \lambda _1&= -(1+2\mu ) + f(\kappa , \mu ), \nonumber \\ \lambda _2&= -(1+2\mu ) - f(\kappa ,\mu ), \, \lambda _3 = -2, \end{aligned}$$

where $f(\kappa ,\mu ) = \frac{\sqrt{\kappa ^4(2\mu -1)^2 - 2\kappa ^3\mu ^2}}{\kappa ^2}$. It is straightforward to show that $L^\pm$ is stable between the lines $\mu =0$ and $\mu +4\kappa =0$ when $\kappa <0$ and unstable between the same lines when $\kappa >0$.

The cases $N_0^\pm , \, N_{\pi }^\pm , \, N_{\pm \frac{\pi }{2}}^{1,2}$, with unequal amplitudes are all unstable where they exist (see Cass 2019 for details).

We plot existence and stability regions for solutions $I, \, A, \, L^\pm , \, N_0^\pm , \, N_{\pi }^\pm$ in Fig. 3. Regions of stable solutions are shown on the left and regions of unstable solutions on the right. Several lines have been labelled, as follows:

37 $$\begin{aligned} HB_A&: \mu =-\frac{1}{2}, \end{aligned}$$

38 $$\begin{aligned} BP_{AL}&: \mu +4\kappa =0, \end{aligned}$$

39 $$\begin{aligned} BP_{II}&: \mu =1, \end{aligned}$$

40 $$\begin{aligned} BP_{AA}&: 3\mu +4\kappa +1=0, \end{aligned}$$

41 $$\begin{aligned} BP_N&: \mu +\kappa =0. \end{aligned}$$

A full explanation of the importance of these lines is given in Appendix B.

We see that bistability and phase-lagging are possible only when $\mu$ and $\kappa$ take different signs. Note the co-existence of stable equal amplitude in-phase *I* and anti-phase *A* synchronisation between the lines $\mu =0, \, \kappa = \frac{1}{8}, \, \mu +4\kappa =0$ in the $(\kappa , \mu )$ plane. Since we assume each of $\gamma , \, \alpha , \, \beta$ is positive, this region in the $(\kappa , \mu )$ plane corresponds to the following region in the (*a*, *b*) coupling plane:

42 $$\begin{aligned} a< 0, \quad b&< \frac{1}{8}(\alpha +3\beta \omega ^2),\nonumber \\&\quad a(\alpha +3\beta \omega ^2)+4\gamma b > 0. \end{aligned}$$

Furthermore, the lines $\mu =0$ and $BP_{AL}$ mark the boundary of stable phase-lagged solutions. The above simple characterisation of these two important behaviours shows the power of our dimensionless analytical approach.

### Steady states for arbitrary parameter values

If we drop the assumption that each of ?, a, $\beta$ is positive, for example, when designing an HDC (Dumas et al. 2014), then it can be shown that (16) becomes

43 $$\begin{aligned}&\dot{R_1} = (s_{\gamma } - s_{\delta }R_1^2)R_1 + (R_1 - R_2 \cos \phi ) \nonumber \\&\quad \times \left( s_{\gamma }\mu + s_{\delta }\kappa (R_1^2 + R_2^2 - 2 R_1 R_2 \cos \phi )\right) , \nonumber \\&\dot{R_2} = (s_{\gamma } - s_{\delta }R_2^2)R_2 + (R_2 - R_1 \cos \phi ) \nonumber \\&\quad \times \left( s_{\gamma }\mu + s_{\delta }\kappa (R_1^2 + R_2^2 - 2 R_1 R_2 \cos \phi )\right) , \nonumber \\&R_1R_2 {\dot{\phi }} = (R_1^2 + R_2^2)\sin \phi \bigl (s_{\gamma }\mu \nonumber \\&\quad + s_{\delta }\kappa (R_1^2 + R_2^2 - 2 R_1 R_2 \cos \phi )\bigr ), \end{aligned}$$

where $s_{\gamma } = \text {sgn}(\gamma ), s_{\delta } = \text {sgn}({\delta })$. Equations (43) have been nondimensionalised using

44 $$\begin{aligned} r_I&= \sqrt{\left| \frac{\gamma }{{\delta }}\right| }, \quad s = \frac{1}{2}|\gamma |t. \end{aligned}$$

There are four cases to consider, according to the sign of $\gamma$, the linear damping coefficient, and the sign of $\delta$, the combined nonlinear damping coefficient (20). Each case leads to a separate stability diagram in the $(\kappa ,\mu )$ plane. Results are given in Appendix C.

In this section, we have extended older work (Leise and Cohen 2007) on the steady states of the aHKB model (16), corresponding to constant amplitude limit cycles, by presenting results in terms of our dimensionless parameters $\kappa , \mu$ (see Table 2). We have also given expressions for bifurcation curves lines $BP_{AL}$, $BP_{II}$, $BP_{AA}$ and $BP_N$ found numerically in recent work (Avitabile et al. 2016).

## Comparison with numerical continuation methods

A detailed bifurcation analysis of (2) using numerical continuation methods. Doedel et al. (1997) was carried out recently (Avitabile et al. 2016). In this section, we compare our analytical results for the aHKB model from Sect. 3 with our own numerical continuation results using AUTO,^6^ for the full HKB model, in order to validate that analysis and determine its region of validity. We will then show how to use our results to predict behaviours in arbitrary parameter regimes. We extend the results in Avitabile et al. (2016) to provide the full picture of the dynamics in the parameter regime considered by these authors, by showing extra solution branches not seen in Avitabile et al. (2016).

### Alternative nondimensionalisation

In Avitabile et al. (2016), the bifurcation analysis was performed with $\gamma \in [-2,8]$ (see Table 1). But with our current nondimensionalisation (15)–(18), $\gamma =0$ corresponds to $\mu$ infinite. So we propose an alternative nondimensionalisation that is better suited for comparison with Avitabile et al. (2016). We define new dimensionless parameters $\nu , \, \sigma$ given by

45 $$\begin{aligned} \nu&\equiv \frac{1}{\mu }=\frac{\gamma }{a}, \end{aligned}$$

46 $$\begin{aligned} \sigma&\equiv \frac{1}{\kappa }=\frac{\delta }{d} = \frac{\alpha +3\beta \omega ^2}{b+3c\omega ^2}. \end{aligned}$$

From (7), we have that

47 $$\begin{aligned} r_I&= 2\sqrt{\frac{\gamma }{\alpha +3\beta \omega ^2}}=\sqrt{\frac{\kappa }{\mu }}\sqrt{\frac{a}{d}}. \end{aligned}$$

Now, we define $\tilde{r}_I$ and $\tilde{s}$ as follows:

48 $$\begin{aligned} \tilde{r}_I&= \sqrt{\frac{a}{d}}=2\sqrt{ \frac{\gamma }{b+3c\omega ^2}}, \end{aligned}$$

49 $$\begin{aligned} \tilde{s}&= \frac{1}{2}|a|t, \end{aligned}$$

and redefine $\tilde{R}_i = \frac{r_i}{\tilde{r}_I}, (i=1,2)$, we find that (43) becomes

50 $$\begin{aligned}&\dot{R_1} = (s_a\nu - s_d\sigma R_1^2)R_1 + (R_1 - R_2 \cos \phi ) \nonumber \\&\quad \times \left( s_a + s_d (R_1^2 + R_2^2 - 2 R_1 R_2 \cos \phi )\right) , \nonumber \\&\dot{R_2} = (s_a\nu - s_d\sigma R_2^2)R_2 + (R_2 - R_1 \cos \phi ) \nonumber \\&\quad \times \left( s_a + s_d(R_1^2 + R_2^2 - 2 R_1 R_2 \cos \phi )\right) , \nonumber \\&R_1R_2 {\dot{\phi }} = (R_1^2 + R_2^2)\sin \phi \bigl (s_a \nonumber \\&\quad + s_d(R_1^2 + R_2^2 - 2 R_1 R_2 \cos \phi )\bigr ), \end{aligned}$$

where we have dropped the tildes over the $R_i$, $s_a = \text {sgn}(a), s_d = \text {sgn}(d)$ and a dot now denotes differentiation with respect to $\tilde{s}$.

### Existence and stability of solutions

We present results for the existence and stability of solutions to (50), corresponding to limit cycles of the aHKB model, as dimensionless parameters $\nu , \sigma$ vary. The methodology is identical to that in Sect. 3. Our aim is to use these results to predict behaviour in an arbitrary range of parameters for the full HKB model (2).

**Fig. 4:**
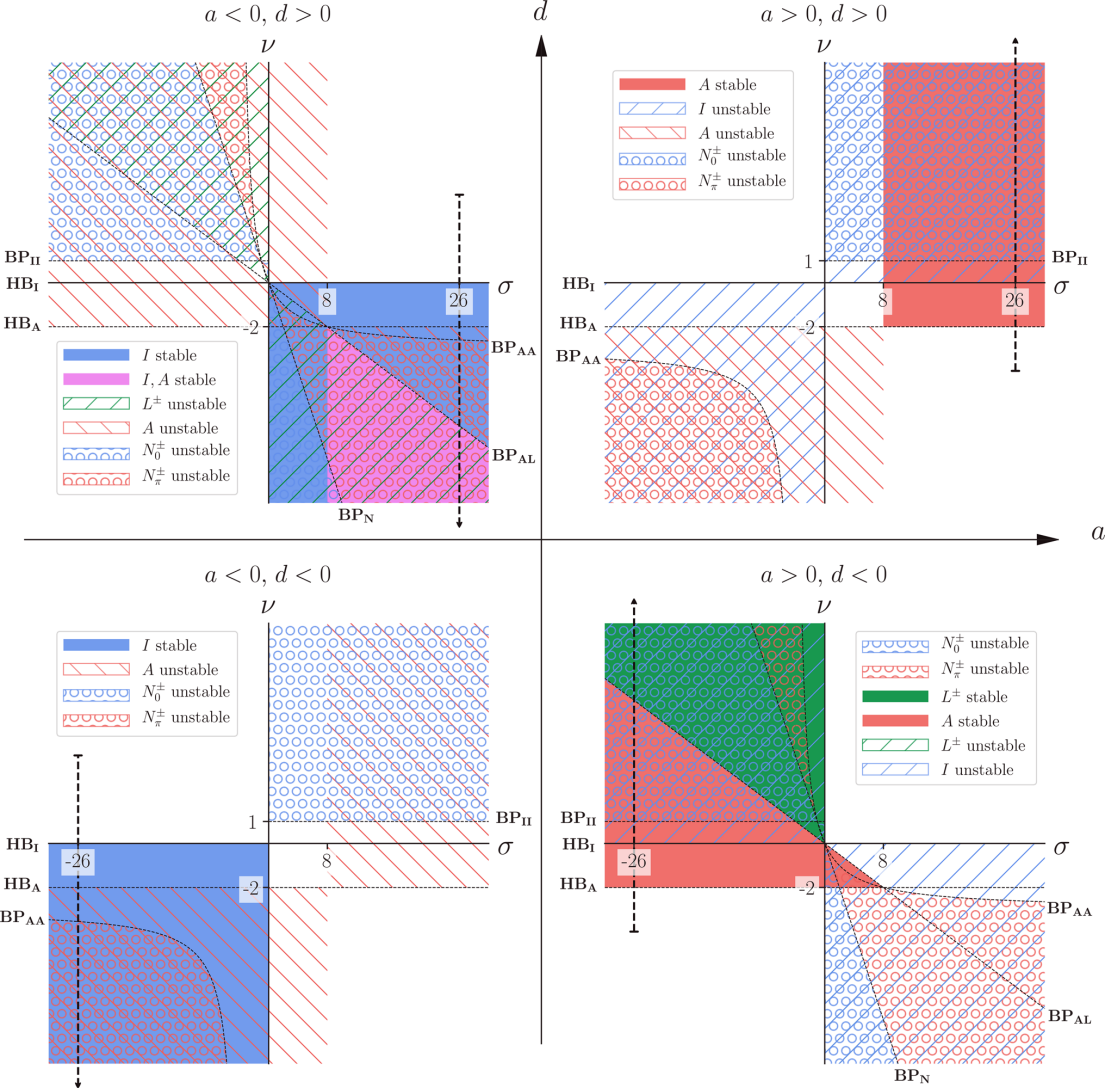
Stability regions of steady states $I, \, A, \, L^\pm , \, N_0^\pm , \, N_{\pi }^\pm$ of (50). The vertical dashed lines correspond to parameter sweeps in the corresponding quadrant of (Avitabile et al. 2016, Figure 4). All curves are labelled according to (37)–(41). This Figure shows how much varied behaviour there is in the approximate aHKB model (16), and how to find it in $(\sigma ,\nu )$-parameter space, for any value of *a*, *d*. We expect the full HKB model (2) to contain even more solutions as the nonlinear terms induce bifurcations. Bear in mind that each point in $(\sigma ,\mu )$ corresponds to many different parameter choices, thus allowing efficient design and development of VPI and HDC

Our results are shown in Fig. 4, arranged in the four quadrants of (*a*, *d*) space.

In Avitabile et al. (2016), results are presented of numerical continuation of the full HKB model (2) for damping parameters $\gamma \in [-2,8], \, \alpha =\beta =1$, coupling parameters $a=\pm 0.5, \, b=\pm 0.5, \, c=0$ and pacing frequency^7^$\omega =2$. From (45), (46), this corresponds to $\nu =\pm 2\gamma , \, \sigma =\pm 26$. Hence, in the first and second quadrants of Fig. 4, we have included a dashed vertical line at $\sigma =26$, and in the third and fourth quadrants, a dashed vertical line at $\sigma =-26$. These lines correspond to parameter values used in (Avitabile et al. 2016, Fig. 4) when $\gamma \in [-2,8]$. We emphasise here that numerical studies of the parameter space are restricted to studying one such line at a time. Along these lines, we use the nondimensional aHKB model (50) to predict what we might find for weakly nonlinear amplitudes in numerical computations of the full HKB model (2).

Figure 4 is possibly the most important figure in this paper. It can be used to predict dynamics along *any* line in $(\sigma , \nu )$ space for arbitrary values of the coupling parameters *a* and *d*.

### Comparison between analysis and numerics

Our analytical results from Sect. 4.2 are valid for weakly nonlinear amplitudes. We should not expect these results to be valid for large amplitudes. To test this, we carried out our own numerical continuations of the full HKB model (2) and plotted these numerical results against our theoretical results from Sect. 3.1, in dimensional form, given in (93)–(97) of Appendix F. Detailed comparisons are given in Appendix D. In summary, we find that

- for small amplitudes, our theoretical results are in complete agreement with our numerical results;
- for intermediate amplitudes, our theoretical results are qualitatively similar to our numerical results, which in turn reveal additional solutions not seen in Avitabile et al. (2016), Fig. 4;

## Discussion and conclusions

The original HKB paper Haken et al. (1985) has attracted considerable scientific attention, owing to its wide applicability in the area of human coordination (see reference in Avitabile et al. 2016).

In this paper, we have provided a fundamental understanding of the HKB model that has significant potential for the development of VPI and HDC.

Our first contribution was to drop all the nonlinear terms from the most widely used form of the HKB model (2) of two coupled oscillators to give the *linear* HKB model (4). A straightforward analysis yields the presence of two system normal modes; in-phase and anti-phase motion with differing stability properties, see Fig. 1.

In turn, the linear HKB model led us to a mechanical analogue (Fig. 2) where the coupling term is seen to be a form of damping between the two subsystems. This analogue leads to the notion that the ratio of linear damping $\gamma$ to linear coupling *a* must be important for the dynamics of the full HKB model and that the in-phase normal mode must be independent of the coupling, whatever form $J(x_1-x_2,{\dot{x}}_1-{\dot{x}}_2)$ takes.

To solve the full HKB model (2), we make use of approximation techniques, part of the method of multiple scales Nayfeh (2008), in which a) any limit cycle has a slowly varying amplitude and b) the linear coefficients $\gamma , \, a \ll \omega$ (the rotating wave approximation). The resulting equations of the approximate HKB (aHKB) model (16) were shown to depend on just two dimensionless parameters: $\mu$, the ratio of linear coupling to linear damping (17) and $\kappa$, the ratio of combined nonlinear coupling to combined nonlinear damping (18).

The discovery that aHKB dynamics is governed by just two parameters has far-reaching consequences. As mentioned above, we know for example that dynamics at $a=a_0, \gamma =\gamma _0$ will be the same as those at $a=ka_0, \gamma =k\gamma _0$ for *any* value of $k \ne 0$. Hence, we can model a variety of different experimental observations using the same model. We could even use results in one experiment to predict behaviour in another.

This applies in particular to phase-lagged results. It is well-known Avitabile et al. (2016) that the relative phase in many real-world applications can take values different from both $0^{\circ }$ (in-phase) and $180^{\circ }$ (anti-phase). For example, a stable relative phase of $90^{\circ }$ is seen in both the amble-to-walk gait of quadrupeds Collins and Stewart (1993) and unsuccessful defences in football Duarte et al. (2012). From Table 2, we see that the general value of the phase-lag is given by $\phi ^* =\pm \cos ^{-1}\left( 1 + \frac{\mu }{2 \kappa }\right)$. So when $\phi ^*=90^{\circ }$, we know that such solutions lie on the line $\mu +2\kappa =0$. We can often estimate $\mu$, so we have a value of $\kappa$ where we can find these $90^{\circ }$ solutions. That value then corresponds (18) to different nonlinear coefficients that we can select, based on the experiment under consideration.

The existence and stability of solutions to the aHKB model (16) were considered in Sect. 3. Some, but not all, of the existence results in that section have been derived earlier Leise and Cohen (2007). But these authors made the restrictive assumption that $\gamma +2a>0$, $\beta >8c$ and $\alpha >8b$, and only worked with the dimensional form of the governing equations.

In Sect. 4, we used the dimensionless approximate HKB model (16) to make predictions about the dynamics of the full HKB model (2). The agreement was excellent for small to moderate amplitudes, as expected, even when $\gamma \sim \omega$. In addition, we discovered additional dynamic behaviour missing from Avitabile et al. (2016). This section contains Fig. 4, possibly the most important figure in the paper.

**Fig. 5:**
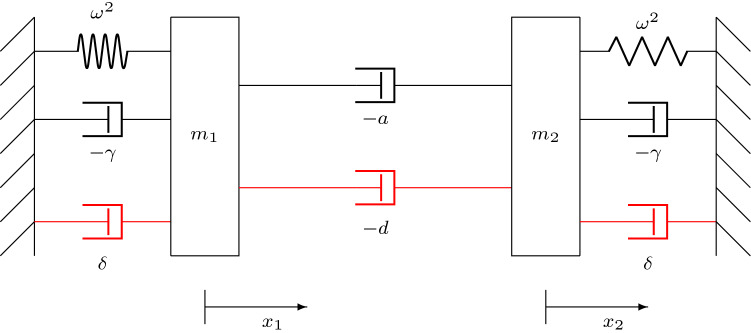
A weakly nonlinear mechanical analogue of the HKB system. This is the same as Fig. 2, except that we have added three weakly nonlinear dampers (shown in red). The extra damping is not directly proportional to the velocity. Instead the coefficients $d, \, \delta$ represent an averaged effect. But since the averaging is the same for these new elements, it is only the ratio $\kappa =\frac{d}{\delta }=\frac{1}{\sigma }$ that matters. This observation is independent of the precise form of HKB model, which is why the weakly nonlinear general HKB model (88) can also be written in terms of two dimensionless parameters. Note that *d* and $\delta$ have opposite signs, since the nonlinear damping term is *softening* for positive parameter values, whereas the nonlinear coupling term is *hardening*

Considering dynamics in $(\kappa , \mu )$ space can be thought of as *weakly nonlinear vs. linear* space. Hence, we propose a *weakly nonlinear* mechanical analogue, shown in Fig. 5.

This is the same as Fig. 2, except that we have added three weakly nonlinear dampers (shown in red). Just as the ratio $\mu =\frac{a}{\gamma }$ is important in the linear mechanical analogue, so the ratio $\kappa =\frac{d}{\delta }$ in the weakly nonlinear analogue is also important. Thus, the relative strength $\mu$ of the linear dampers is complemented by the relative strength $\kappa$ of the weakly nonlinear dampers.

One feature that can explained using Fig. 5 is the point $(\mu , \kappa ) = (1, -1)$, $[(\nu , \sigma ) = (1, -1)]$, visible in Fig. 3 (right hand side) and Fig. 4. This occurs when $BP_N$, $BP_{II}$, and $BP_{AA}$ all intersect. In dimensional terms, this point corresponds to $\gamma =a$ (the linear damping and coupling coefficients being equal) and $d=-\delta$ (this happens when the combined nonlinear corrections to damping and coupling are equal). At this point, the weakly nonlinear damping and coupling are identical.

As mentioned in Sect. 2, much work has been done in establishing the correct form of $h(x,{\dot{x}})$ and $J(x_1-x_2,{\dot{x}}_1-{\dot{x}}_2)$ in (1). In their original paper Haken et al. (1985), the authors described establishing the form of the coupling term $J(x_1-x_2,{\dot{x}}_1-{\dot{x}}_2)$ as “...*the central problem, namely to derive a suitable coupling between* ...$x_1$ and $x_2$.”

We can now consider their two other choices within the framework of our mechanical analogue. The first choice (Haken et al. 1985, equation (3.11a)) was $J(x_1-x_2,{\dot{x}}_1-{\dot{x}}_2) = [a+b(x_1-x_2)^2](x_1-x_2)$. When $b=0$, this corresponds to replacing the central dashpot in Fig. 2 with a spring of stiffness *a*. When $b \ne 0$, this corresponds to replacing both the central dashpot with a spring of stiffness *a* and the central red weakly nonlinear dashpot with a weakly nonlinear spring of stiffness $a+3b\omega ^2$. This choice of $J(x_1-x_2,{\dot{x}}_1-{\dot{x}}_2)$ was rejected because both linear and weakly nonlinear forms failed to produce the correct type of equation for ${\dot{\phi }}$. The second choice of (Haken et al. 1985, equation (3.11b)) added a time delay to the first choice, in an integral sense that became ${\dot{J}}(x_1-x_2,{\dot{x}}_1-{\dot{x}}_2) = [a+b(x_1-x_2)^2](x_1-x_2)$ under approximation. So we can modify our analogue accordingly to include mechanical elements that integrate resistance over time. This second form of $J(x_1-x_2,{\dot{x}}_1-{\dot{x}}_2)$ did produce the correct relationship between $x_1$ and $x_2$ but was not considered further by the authors.

In Appendix E, we show that a very general form of the aHKB equation (88) can be represented by one set of nondimensionalised equations (16) involving only dimensionless parameters $\mu$, already given in (17), and a redefined $\kappa$, in (90). This can be expected from Fig. 5, where the precise form of the HKB equation is not important. These more general models may be of use to the VPI/HDC community in choosing models of coordination for experiments.

We end with some general observations about the form of the HKB model (1). The methods of nonlinear dynamics (including numerical continuation) are far more powerful than the weakly nonlinear techniques used to study the HKB equations to date. But the new solutions found by these methods are not just of passing interest. They are present in the model and so should be seen in experiments. We hope that experiments will soon be performed to test out these predictions.

But, absent any experimental evidence, another possibility is that the HKB model itself needs revision. The original authors themselves already suggested another acceptable form of $J(x_1-x_2,{\dot{x}}_1-{\dot{x}}_2)$, as we have just mentioned. Does that contain any of the solutions we see here? Then, there are the extensions to the HKB model that explicitly include time delays Banerjee and Jirsa (2006), Słowiński et al. (2016), Słowiński et al. (2020). But these seem to include even more behaviour than seen in experiments to date. Perhaps one place to start is if we ask the question posed by the linear HKB model (4) and its mechanical analogue, Fig. 2. What is the physiological meaning of the damping that connects the two oscillators?

## Figures and Tables

**Fig. 6:**
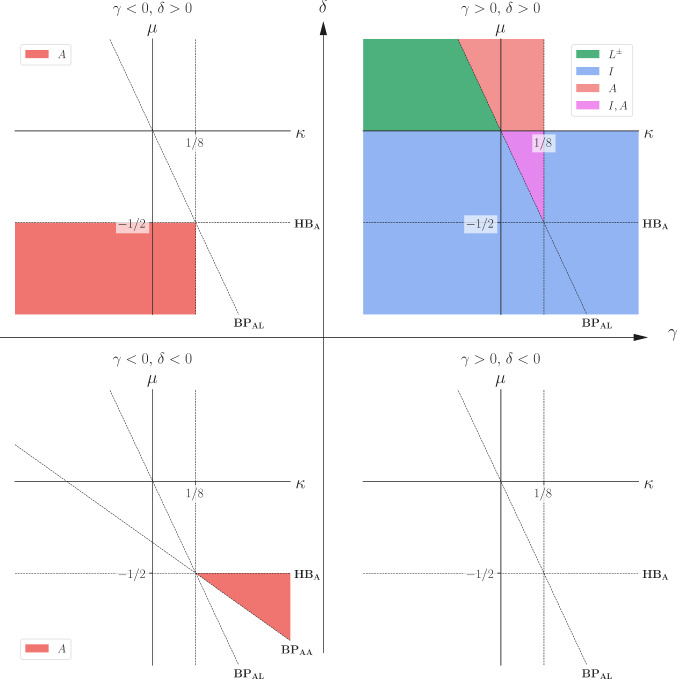
Stability regions of the three cases of equal amplitude synchronisation $I, \, A, \, L^{\pm }$. There are four separate stability diagrams in the $(\kappa ,\mu )$ plane, according to the signs of $\gamma , \, \delta$. The plot in the first quadrant is identical to the left hand figure in Fig. 3

**Fig. 7:**
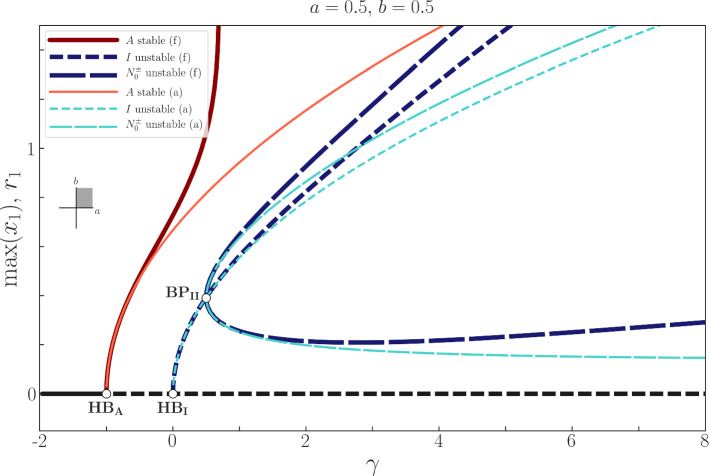
Bifurcation diagram in $\gamma$ computed for coupling strengths $a=0.5, \, b=0.5$. The thicker solid/dashed lines represent stable/unstable states of the full (f) HKB model (2), obtained using our own numerical methods. Overlaid on each figure, using thinner lines, are our analytic results from Sect. 3 of the approximate (a) aHKB model in dimensional form (93)–(97). Compare these numerical results with the theoretical predictions in the first quadrant of Fig. 4 along the line $\sigma =26$, together with Table 3. The solution branches that emerge from $BP_{II}$ are $I, N^{\pm }_0$; all three are unstable. The upper branch $N^+_0$ is missing from Avitabile et al. (2016), Fig. 4

**Fig. 8:**
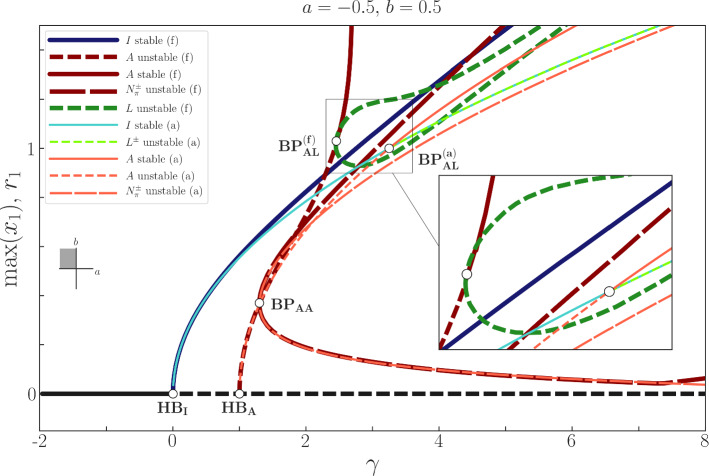
Bifurcation diagram for coupling strengths $a= -0.5, \, b= 0.5$, to be compared with predictions in the second quadrant of Fig. 4 along the line $\sigma =26$, together with Table 3. The solution branches that emerge from $BP_{AA}$ are $A, N^{\pm }_{\pi }$; all three are unstable. The upper branch $N^+_{\pi }$ is missing from (Avitabile et al. 2016, Figure 4), as is the lower branch of *unstable* solutions that emerge from $BP_{AL}^{(f)}$. Our analysis of the aHKB model predicts a bifurcation $BP_{AL}^{(a)}$ at $\gamma =\frac{13}{4}=3.25$ for these parameter values (see Table 3). At $BP_{AL}^{(a)}$, stable *I* and unstable *A* collide to give rise to stable $I, \, A$ and unstable *equal* amplitude phase-lagged solutions $L^{\pm }$ (see inset for more detail) Note that $I, \, L^{\pm }$ have the same amplitude at this level of approximation and so the solution branches are indistinguishable

**Fig. 9:**
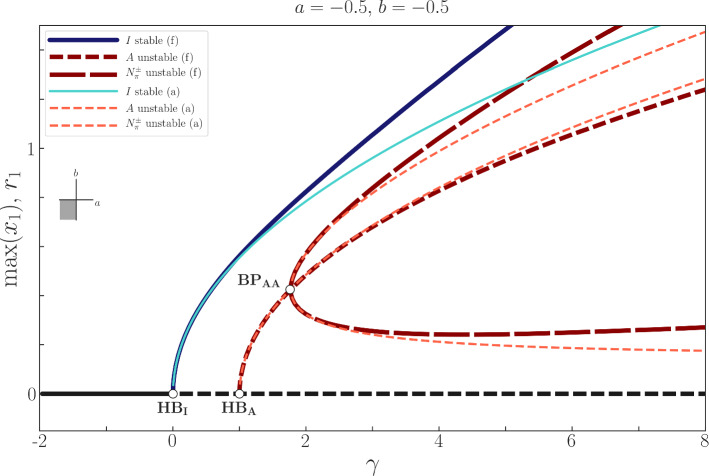
Bifurcation diagram for coupling strengths $a= -0.5, \, b= -0.5$, to be compared with predictions in the third quadrant of Figure 4, along the line $\sigma =-26$, together with Table 3. The solution branches that emerge from $BP_{AA}$ are $A, N^{\pm }_{\pi }$; all three are unstable. The lower branch $N^-_{\pi }$ is missing from (Avitabile et al. 2016, Figure 4)

**Fig. 10:**
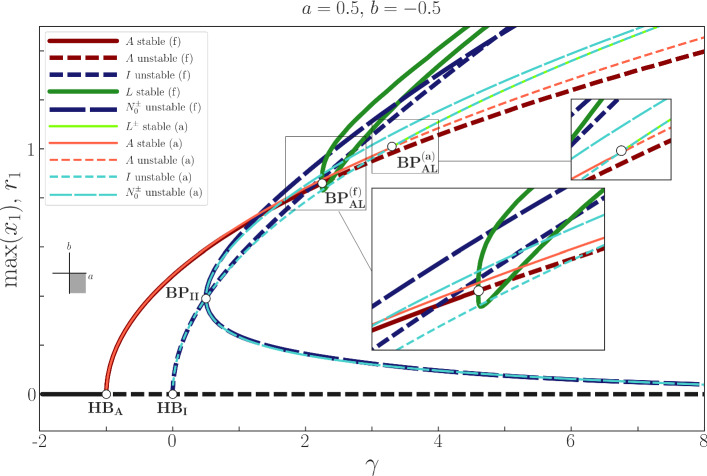
Bifurcation diagram for coupling strengths $a= 0.5, \, b= -0.5$ to be compared with predictions in the fourth quadrant of Figure 4 along the line $\sigma =-26$, together with Table 3. The solution branches that emerge from $BP_{II}$ are $I, N^{\pm }_0$; all three are unstable. The lower branch $N^-_0$ is missing from (Avitabile et al. 2016, Figure 4), as is the lower branch of *stable* solutions that emerge from $BP_{AL}^{(f)}$. Our analysis of the aHKB model predicts a bifurcation $BP_{AL}^{(a)}$ at $\gamma =\frac{13}{4}=3.25$ for these parameter values (see Table 3). At $BP_{AL}^{(a)}$, unstable *I* and stable *A* collide to give rise to unstable $I, \, A$ and stable equal amplitude phase-lagged synchronisation $L^{\pm }$ (see inset for more detail). Note that $I, \, L^{\pm }$ have the same amplitude at this level of approximation and so the solution branches are indistinguishable

## Data Availability

(data transparency): Not applicable

## A Normal modes

For in-phase motion $x_1=x_2$, and we have $\eta _A =0$; for anti-phase motion $x_1=-x_2$, we have $\eta _I =0$. Note the asymmetry between the dynamics of $\eta _{I,A}$ in (5). The normal modes $\eta _{I,A}$ are identical to the symmetric and anti-symmetric coordinates $\psi _{\pm }$ of Fuchs and Jirsa (2000). But the connection with normal modes was not made in that paper. From (5), if $\gamma >0$, the in-phase normal mode $\eta _I$ is always unstable. Then, (i) if $\gamma + 2 a >0$, both normal modes $\eta _{I,A}$ are unstable; (ii) if $2a +\gamma <0$, the anti-phase normal mode $\eta _A$ is stable. If $\gamma <0$, the in-phase normal mode $\eta _I$ in (5) is always stable. Then, (iii) if $\gamma + 2 a >0$, $\eta _A$ is unstable—this happens because the coupling coefficient *a* is strong enough to overcome the damping, leading to instability; (iii) if $2a +\gamma <0$, then $\eta _A$ is stable and we would not expect to see any form of limit cycle in the full HKB model (2) in this region of parameter space. These four cases are shown in Fig. 1. From Table 1, we observe that nearly all studies of the full HKB model (2) are carried out in the fourth quadrant, where $\gamma >0,\, a<0$. The dynamics within the regions of Fig. 1 are as follows. From (5), we have

51 $$\begin{aligned} \eta _I&= C^+_Ie^{\lambda ^+_It} + C^-_Ie^{\lambda ^-_It}, \nonumber \\ \eta _A&= C^+_Ae^{\lambda ^+_At} + C^-_Ae^{\lambda ^-_At}, \end{aligned}$$

where $C^{\pm }_{I,A}$ are constants and

52 $$\begin{aligned} \lambda ^{\pm }_I&= \frac{1}{2} (\gamma \pm i\sqrt{|4\omega ^2-\gamma ^2|}), \nonumber \\ \lambda ^{\pm }_A&= \frac{1}{2} ((2a+\gamma ) \pm i\sqrt{|4\omega ^2-(2a+\gamma )^2|}), \end{aligned}$$

where we have assumed that $\gamma , a \ll \omega$. Since $x_1=\frac{1}{2}(\eta _I+\eta _A)$ and $x_2=\frac{1}{2}(\eta _I-\eta _A)$, we see that $x_{1,2}$ are both stable in *S*, but at least one of them is unstable elsewhere.

## B Bifurcations

In Figs. 3–10, we labelled certain curves, following the convention in Avitabile et al. (2016). We can now explain their role in our approximate analysis and the relationship with the numerical solutions. Expressions for them in terms of both pairs of dimensionless parameters $(\kappa , \mu )$ and $(\sigma ,\nu )$ and in terms of the system parameters of (2) with $c=0$ are given in Table 3.

Curves HB_I,A_, BP_II,AA_ appear in all four quadrants in Fig. 4 and appear to have identical roles in both analysis and computations:

- HB_I_: Hopf bifurcation of the equal amplitude in-phase solution *I*. It is well-known in the literature of the HKB model and occurs when the linear damping term $\gamma$ changes sign.
- HB_A_: Hopf bifurcation of the equal amplitude anti-phase solution *A*. It occurs when the linear damping term $2a+\gamma$ of the anti-phase normal mode $\eta _A$ (5) changes sign.
- BP_II_: Symmetry-breaking bifurcation of the equal amplitude in-phase solution *I*.
- BP_AA_: Symmetry-breaking bifurcation of the equal amplitude anti-phase synchronisation *A*.

Curve BP_AL_: $4\nu +\sigma =0$ appears in Fig. 4, where it has two sets of four different roles in the second and fourth quadrants. We have seen it, labelled $BP_{AL}^{(a)}$, in one role in Fig. 8 and in another role in Fig. 10. The numerical equivalent of these roles is labelled $BP^{(f)}_{AL}$ in the same figures.

Curve BP_N_: $\nu +\sigma =0$ separates $N^{\pm }_0$ solutions from $N^{\pm }_{\pi }$ solutions. It features in Fig. 4, where it has a number of roles in the second and fourth quadrants. We do not see it in Figs. 7–10 since it occurs at very large values of $\gamma$.

Curve BP_IL_ appears in (Avitabile et al. 2016, Figure 5(a)), as the line $a=0$. It corresponds to a phase bifurcation between equal amplitude in-phase synchronisation *I* and equal amplitude phase-lagged synchronisation $L^{\pm }$.

Finally, we mention the point FH (a fold-Hopf bifurcation) that appears in (Avitabile et al. 2016, Figure 5(b)).It occurs around $\gamma =-1, \, b\approx 1.625$ when $a=0.5$ (Avitabile et al. 2016, p. 209). In our analysis, this corresponds to the point $(\kappa ,\mu )=(\frac{1}{8},-\frac{1}{2})$ [ $(\sigma ,\nu )=(8,-2)$, or $(a,b)=(-\frac{1}{2}\gamma ,\frac{1}{8}(\alpha +3\beta \omega ^2))$]. When $a=0.5$, we have $\gamma =-1$, and substituting in the relevant parameter values, we predict that FH occurs at $b=\frac{13}{8}=1.625$, very close to the stated numerical value Avitabile et al. (2016). In Fig. 3, FH can be seen as the intersection of the three lines $HB_A$, $BP_{AA}$ and $BP_{AL}$. FH also appears in the second and fourth quadrants of Fig. 4. In our numerical computations, FH would correspond to varying parameters $a, \, b$ in Fig. 8 so that the bifurcation points $HB_A$, $BP_{AA}$ and $BP^{(a)}_{AL}$ all coincide.

## C Different cases

If we drop the assumption that each of $\gamma , \, \alpha , \, \beta$ is positive, then it can be shown that the aHKB model (13) takes on a new form given by (43). There are four cases to consider, according to the signs of $\gamma$ and $\delta$ (20).

The results for stable solutions are shown in Fig. 6, where only the three cases of equal amplitude synchronisation $I, \, A, \, L^{\pm }$ appear.

The plot in the first quadrant of Fig. 6 is identical to the left hand figure in Fig. 3, since we assumed $\gamma >0$ and taking $\alpha , \beta >0$ ensures that $\delta =\frac{1}{4}(\alpha +3\beta \omega ^2)>0$.

In the second and third quadrants of Fig. 6, we have $\gamma < 0$. We find regions of stable equal amplitude anti-phase synchronisation *A* for all nonzero values of $\delta$, as can be expected from the analysis in Sect. 2.1.

Note that there is no stable equal amplitude synchronisation $I, \, A, \, L^{\pm }$ in the fourth quadrant of Fig. 6, where $\gamma >0, \, \delta <0$. So all stable synchronisation for $\gamma >0$ are captured in the first quadrant, corresponding to parameter values used in most experimental studies of the HKB model (see Table 1).

## D Comparison

In what follows, we organise our results according to the quadrants of (Avitabile et al. 2016, Fig. 4), which correspond directly to those in our own Fig. 4. Throughout this comparison, we set $\alpha =1, \, \beta =1$, $\omega =2$.

### D.1 First quadrant: $a=0.5, \, b=0.5$

Figure 7 is a bifurcation diagram of $max(x_1), \, r_1$ vs. $\gamma$, computed for coupling strengths $a= 0.5, \, b= 0.5$, using AUTO Doedel et al. (1997),    where $r_1$ is defined in (12). Thethicker lines represent numerical computations of the full (f) HKB model (2): solid lines are stable solutions, dashed lines are unstable solutions. Overlaid on each figure, using thinner lines, are the dimensional forms of our analytic results (93)–(97) of the approximate (a) aHKB model, given in Appendix F. We compare these results with the analytic predictions in the first quadrant of Fig. 4, where $d=\frac{1}{4}b>0$, along the line $\sigma =26$, together with Table 3.

**Table 3:** Analytic expressions for features seen in Figs. 3–10 and (Avitabile et al. 2016, Fig. 4), in terms of both pairs of dimensionless parameters $(\kappa , \mu )$ and $(\sigma ,\nu )$, and in terms of the system parameters $\gamma ,\, \alpha ,\, \beta ,\, a,\, b,\, \omega$. The expressions involving system parameters are obtained by undoing the scaling and setting $c=0$

Label	$(\kappa , \mu )$	$(\sigma ,\nu )$	System parameters of (2) with $c=0$
HB_I_	$\mu$ infinite	$\nu =0$	$\gamma =0$
HB_A_	$\mu =-\frac{1}{2}$	$\nu =-2$	$2a+\gamma =0$
BP_II_	$\mu =1$	$\nu =1$	$a-\gamma =0$
BP_AA_	$3\mu +4\kappa +1=0$	$\nu (\sigma +4)+3\sigma =0$	$3a(\alpha +3\beta \omega ^2)+4\gamma b +\gamma (\alpha +3\beta \omega ^2)=0$
BP_AL_	$\mu +4\kappa =0$	$4\nu +\sigma =0$	$a(\alpha +3\beta \omega ^2)+4\gamma b=0$
BP_N_	$\mu +\kappa =0$	$\nu +\sigma =0$	$a(\alpha +3\beta \omega ^2)+b\gamma =0$
BP_IL_	$\mu =0$	$\nu$ infinite	$a=0$
FH	$(\kappa ,\mu )=(\frac{1}{8},-\frac{1}{2})$	$(\sigma ,\nu )=(8,-2)$	$(a,b)=(-\frac{1}{2}\gamma ,\frac{1}{8}(\alpha +3\beta \omega ^2))$

### D.2 Second quadrant: $a=-0.5, \, b=0.5$

Figure 8 is a bifurcation diagram for coupling strengths $a= -0.5,\, b= 0.5$, to be compared with predictions in the second quadrant of Fig. 4, along the line $\sigma =26$, together with Table 3. Although details are slightly different, both our theory and numerics predict that stable $I, \, A$ can co-exist in the presence of unstable phase-lagged motion.

### D.3 Third quadrant: $a=-0.5, \, b=-0.5$

Figure 9 is a bifurcation diagram for coupling strengths $a= -0.5, \, b= -0.5$, to be compared with predictions in the third quadrant of Figure 4, along the line $\sigma =-26$, together with Table 3.

### D.4 Fourth quadrant: $a=0.5, \, b=-0.5$

Figure 10 is a bifurcation diagram for coupling strengths $a= 0.5, \, b= -0.5$, to be compared with predictions in the fourth quadrant of Figure 4, along the line $\sigma =-26$, together with Table 3. Although details are slightly different, both our theory and our numerics predict that stable *A* loses stability and gives rise to stable phase-lagged motion.

## E Generalised HKB model

We show that the nondimensional version of the aHKB equation (16) holds for quite general forms of damping and coupling, subject only to redefinition of $r_I$ (89) and $\kappa$ (90). The rescaled time *s* (15) and parameter $\mu$ (17) remain the same.

### E.1 Generalised nonlinear damping

To begin with, let us consider a general nonlinear damping term of the form

53 $$\begin{aligned} \beta |x|^{2-2p}|{\dot{x}}|^{2p}{\dot{x}}, \end{aligned}$$

for $p \in [0,1]$. The full HKB model (2) contains two such terms: the Rayleigh and Van der Pol terms corresponding to $p=1$ and $p=0$, respectively.

Before considering two coupled oscillators, let us demonstrate our approach by considering a single uncoupled oscillator, with one general damping term, of the form

54 $$\begin{aligned} \ddot{x} + \omega ^2 x = \epsilon (\gamma - \beta |x|^{2-2p}|{\dot{x}}|^{2p}){\dot{x}}. \end{aligned}$$

We examine the dynamics of (54), using two-timing Cass (2019). We give some details of our working, since the elimination of secular terms is more involved than outlined in Sect. 2.4. Taking $x(t,\epsilon ) = x^0(\tau ,T) + \epsilon x^1(\tau ,T) + O(\epsilon ^2)$ as in (11), we have

55 $$\begin{aligned} O(1): \quad \ddot{x}^0 + \omega ^2 x^0 =&0, \end{aligned}$$

56 $$\begin{aligned} O(\epsilon ): \quad \ddot{x}^1 + \omega ^2 x^1 =&- 2\partial _\tau \partial _T x^0 + \gamma \partial _\tau x^0 \nonumber \\&- \beta |x^0|^{2-2p}|\partial _\tau x^0|^{2p}. \end{aligned}$$

Hence from (55), we have

57 $$\begin{aligned} x^0(\tau , T)&= r(T) \cos (\omega \tau + \phi (T)) \end{aligned}$$

58 $$\begin{aligned}&= \frac{1}{2}(A(T)e^{i\omega \tau }+A^*(T)e^{-i\omega \tau }), \end{aligned}$$

corresponding to a limit cycle of slowly varying amplitude *r*(*T*) and phase $\phi (T)$, where

59 $$\begin{aligned} A(T)&= \frac{1}{2}r(T)e^{i \phi (T)} \end{aligned}$$

is the complex amplitude.

The next step is to substitute (57) into the right hand side of (56). Let $f(\tau ) \equiv - \beta |x^0|^{2-2p}|\partial _\tau x^0|^{2p}$. Then using (57), we find

60 $$\begin{aligned} f(\tau )&= \beta \omega ^{2p + 1} r^3|\cos (\omega \tau + \phi )|^{2-2p} \nonumber \\&\quad \times |\sin (\omega \tau + \phi )|^{2p} \sin (\omega \tau + \phi ). \end{aligned}$$

To find the secular terms in $f(\tau )$ at $O(\epsilon )$, we calculate the complex Fourier series expansion of $f(\tau )$. The coefficient $c_1$ of $e^{i \omega \tau }$ in this expansion is given by $c_1 = \frac{1}{2}(a_1 - i b_1)$, where

61 $$\begin{aligned} a_1&= \frac{2}{T}\int _0^T f(\tau ) \cos \omega \tau \, \mathrm{d}\tau , \nonumber \\ b_1&= \frac{2}{T}\int _0^T f(\tau ) \sin \omega \tau \, \mathrm{d}\tau . \end{aligned}$$

Setting $\theta = \omega \tau + \phi$ and substituting (60) into (61), we find

62 $$\begin{aligned} a_1&= \frac{\beta \omega ^{2p+1} r^3}{\pi }\int _0^{2 \pi } |\cos \theta |^{2-2p} \nonumber \\&\quad \times |\sin \theta |^{2p} \sin \theta \cos (\theta - \phi )\, \mathrm{d}\theta . \end{aligned}$$

The expansion $\cos (\theta - \phi )=\cos \theta \cos \phi +\sin \theta \sin \phi$ then leads to two integrals on the right hand side of (62). The first integral vanishes, since the integrand is an odd function. The second integral can be simplified to become

63 $$\begin{aligned} a_1 = \frac{4 \beta \omega ^{2p+1} r^3 \sin \phi }{\pi }\int _0^{\frac{\pi }{2}} \cos ^{2-2p}(\theta )\sin ^{2+2p}(\theta ) \, \mathrm{d}\theta . \end{aligned}$$

Then, using the identity

64 $$\begin{aligned} \int _0^{\frac{\pi }{2}} \cos ^{2s-1}\theta \sin ^{2z-1}\theta \, \mathrm{d}\theta = \frac{1}{2}\frac{\varGamma (s)\varGamma (z)}{\varGamma (s+z)}, \end{aligned}$$

we have

65 $$\begin{aligned} a_1 = g(\beta ,p) \omega r^3 \sin \phi , \end{aligned}$$

where

66 $$\begin{aligned} g(\beta ,p) \equiv \frac{1}{\pi }\varGamma \left( \frac{3}{2}-p\right) \varGamma \left( \frac{3}{2}+p\right) \beta \omega ^{2p}, \end{aligned}$$

Similarly, from (61) we find

67 $$\begin{aligned} b_1 = g(\beta ,p) \omega r^3 \cos \phi , \end{aligned}$$

and hence

68 $$\begin{aligned} c_1 = \frac{1}{2}(a_1 - i b_1)&= \frac{1}{2}g(\beta ,p) \omega r^3 (\sin \phi - i\cos \phi ) \nonumber \\&= -4ig(\beta ,p)\omega |A|^2 A \end{aligned}$$

where the complex amplitude *A* is defined in (59).

We then obtain an evolution equation for *A*(*T*) by setting to zero the secular terms in (56) to find

69 $$\begin{aligned} 2 {\dot{A}} = \left[ \gamma - 4g(\beta ,p)|A|^2\right] A, \end{aligned}$$

and hence

70 $$\begin{aligned} {\dot{r}}&= \frac{1}{2}\left[ \gamma - g(\beta ,p) r^2\right] r, \end{aligned}$$

71 $$\begin{aligned} {\dot{\phi }}&= 0. \end{aligned}$$

The amplitude of the resulting limit cycle is therefore

72 $$\begin{aligned} r_I = \sqrt{\frac{\gamma }{g(\beta , p)}}. \end{aligned}$$

We can compare this result with (7), since the equation for a single oscillator is identical to the equation for equal amplitude in-phase oscillations (see Sect. 2.3). For the Van der Pol oscillator, we have $p=0$ and $\beta =\alpha$. Hence, from (66), $g(\alpha ,0) =\frac{1}{\pi }\varGamma ^2(\frac{3}{2})\alpha =\frac{1}{4}\alpha$ since $\varGamma (\frac{3}{2})=\frac{1}{2}\sqrt{\pi }$. So from (72), we have $r_I = \sqrt{\frac{\gamma }{g(\alpha , 0)}}=2\sqrt{\frac{\gamma }{\alpha }}$ in agreement with (7) when $\beta =0$.

For the Rayleigh oscillator, we have $p=1$. Hence, from (66), $g(\beta ,1)= \frac{1}{\pi }\varGamma (\frac{1}{2})\varGamma (\frac{5}{2})\beta \omega ^{2}=\frac{3}{4}\beta \omega ^2$, since $\varGamma (\frac{1}{2})=\sqrt{\pi }$ and $\varGamma (\frac{5}{2})=\frac{3}{4}\sqrt{\pi }$. So from (72), we have $r_I = \sqrt{\frac{\gamma }{g(\beta , 1)}}=2\sqrt{\frac{\gamma }{3\beta \omega ^2}}$ in agreement with (7) when $\alpha =0$.

Now, consider a single general hybrid oscillator with *n* general nonlinear damping terms of the form (53):

73 $$\begin{aligned} \ddot{x} + \omega ^2 x = \epsilon \left[ \gamma - \sum _{i=1}^{n} \beta _i |x|^{2-2p_i}|{\dot{x}}|^{2p_i}\right] {\dot{x}}, \end{aligned}$$

where $p_i \in [0,1], \; i=1\ldots n$ and $\beta _i, \; 1=1\ldots n$ are damping coefficients.

The effect of these extra nonlinear terms is additive, so that the amplitude equation (70) generalises to

74 $$\begin{aligned} {\dot{r}} = \frac{1}{2}\left[ \gamma - \sum _{i=1}^n g(\beta _i, p_i) \, r^2 \right] r, \end{aligned}$$

and the resulting limit cycle amplitude becomes

75 $$\begin{aligned} r_I = \sqrt{\frac{\gamma }{\sum _{i=1}^n g(\beta _i, p_i)}}. \end{aligned}$$

Equation (75) is the amplitude of the limit cycle that occurs in a single general hybrid oscillator, subject to *n* general nonlinear damping terms of the form (53). When $n=2, \, \beta = \alpha , \, p_1=0, \beta _2=\beta , \, p_2=1$, (75) agrees with (7).

### E.2 Generalised nonlinear coupling

Now, we consider generalised nonlinear coupling of the form

76 $$\begin{aligned} b({\dot{x}}_1-{\dot{x}}_2)|x_1 - x_2|^{2-2q}|{\dot{x}}_1 - {\dot{x}}_2|^{2q}, \end{aligned}$$

where $q \in [0,1]$. The full HKB model (2) contains two such terms, one with $q=0$ and the other with $q=1$.

To demonstrate how to handle the secular terms in this case, we consider Rayleigh damping only. Our equations become:

77 $$\begin{aligned} \ddot{x}_1 + \omega ^2 x_1&= \epsilon \bigl [(\gamma - \beta {\dot{x}}_1^2){\dot{x}}_1 + ({\dot{x}}_1-{\dot{x}}_2) \nonumber \\&\quad \times (a + b|x_1 - x_2|^{2-2q}|{\dot{x}}_1 - {\dot{x}}_2|^{2q})\bigr ], \nonumber \\ \ddot{x}_2 + \omega ^2 x_2&= \epsilon \bigl [(\gamma - \beta {\dot{x}}_2^2){\dot{x}}_2 + ({\dot{x}}_2-{\dot{x}}_1) \nonumber \\&\quad \times (a + b|x_2 - x_1|^{2-2q}|{\dot{x}}_2 - {\dot{x}}_1|^{2q})\bigr ]. \end{aligned}$$

Using two timing Cass (2019), we set $x_i(t,\epsilon ) = x_i^0(\tau ,T) + \epsilon x_i^1(\tau ,T) + O(\epsilon ^2), \, (i=1,2)$, where $\tau =t, T=\epsilon t$. Then, the generalised coupling term (76) contribution at $O(\epsilon )$ is

78 $$\begin{aligned} b(\partial _\tau x_1^0 - \partial _\tau x_1^1)|x_1^0 - x_2^0|^{2-2q}|\partial _\tau x_1^0 - \partial _\tau x_1^1|^{2q}. \end{aligned}$$

Setting $x_i^0(\tau ,T) = r_i (T) \cos (\omega \tau + \phi _i(T)), \, (i=1,2)$ and $A_i(T) = \frac{1}{2}r_i(T)e^{i \phi _i(T)}, \, (i=1,2)$, (78) becomes

79 $$\begin{aligned}&b(r_2 \omega \sin (\omega \tau + \phi _2) - r_1 \omega \sin (\omega \tau + \phi _1)) \nonumber \\&\quad \times |r_1 \cos (\omega \tau + \phi _1) - r_2 \cos (\omega \tau + \phi _2)|^{2-2q}\nonumber \\&\quad \times |r_2 \omega \sin (\omega \tau + \phi _2) - r_1 \omega \sin (\omega \tau + \phi _1)|^{2q}. \end{aligned}$$

Now, we define new variables ${\hat{R}}$ and $\varPhi$ as follows

80 $$\begin{aligned} {\hat{R}} \sin \varPhi&= r_1 \sin \phi _1 - r_2 \sin \phi _2, \nonumber \\ {\hat{R}} \cos \varPhi&= r_1 \cos \phi _1 - r_2 \cos \phi _2. \end{aligned}$$

Hence, ${\hat{R}}^2 = (r_1^2 + r_2^2 - 2 r_1 r_2 \cos \phi )$. Equation (79) then reduces to

81 $$\begin{aligned} -b \omega ^{2q+1}{\hat{R}}^3|\cos (\omega \tau + \varPhi )|^{2-2q}|\sin (\omega \tau + \varPhi )|^{2q}\sin (\omega \tau + \varPhi ),\nonumber \\ \end{aligned}$$

which has exactly the same form as (60). Hence, we can immediately write down expressions for the Fourier coefficients $a_1,b_1$, as defined in (61), to find

82 $$\begin{aligned} a_1 = -g(b, q) \, \omega {\hat{R}}^3 \sin \varPhi , \quad b_1 = -g(b,q) \, \omega {\hat{R}}^3 \cos \varPhi . \end{aligned}$$

where the function $g(\cdot ,\cdot )$ is given in (66). Noting that

83 $$\begin{aligned} {\hat{R}}^3 \sin \varPhi&= (r_1^2 + r_2^2 - 2 r_1 r_2 \cos \phi ) \nonumber \\&\quad \times (r_1 \sin \phi _1 - r_2 \sin \phi _2) \nonumber \\ {\hat{R}}^3 \cos \varPhi&= (r_1^2 + r_2^2 - 2 r_1 r_2 \cos \phi ) \nonumber \\&\quad \times (r_1 \cos \phi _1 - r_2 \cos \phi _2), \end{aligned}$$

the coefficient of $e^{i \omega \tau }$ in the complex Fourier series is thus

84 $$\begin{aligned} c_1&= \frac{1}{2}(a_1 - i b_1) \nonumber \\&= -\frac{1}{2} \, g(b,q) \, \omega (r_1^2 + r_2^2 - 2 r_1 r_2 \cos \phi ) \nonumber \\&\quad \times [(r_1 \sin \phi _1 - r_2 \sin \phi _2)-i(r_1 \cos \phi _1 - r_2 \cos \phi _2)] \nonumber \\&= \frac{1}{2} \, g(b,q) \, i \omega (r_1^2 + r_2^2 - 2 r_1 r_2 \cos \phi )(r_1 e^{i \phi _1} - r_2 e^{i \phi _2})\nonumber \\&= -4 \, g(b,q) \, i \omega |A_2 - A_1|^2(A_2 - A_1). \end{aligned}$$

Hence, the conditions to eliminate secular terms at $O(\epsilon )$ are, in terms of the complex amplitudes $A_1, A_2$,

85 $$\begin{aligned} 2 {\dot{A}}_1&= \left( \gamma - 3 \beta \omega ^2|A_1|^2\right) A_1 \nonumber \\&\quad + (a + 4\, g(b,q) \,|A_1 - A_2|^2)(A_1 - A_2), \nonumber \\ 2 {\dot{A}}_2&= \left( \gamma - 3 \beta \omega ^2|A_2|^2\right) A_2 \nonumber \\&\quad + (a + 4\, g(b,q) \,|A_2 - A_1|^2)(A_2 - A_1), \end{aligned}$$

and, in terms of the real amplitudes $r_1, r_2$ and relative phase $\phi$

86 $$\begin{aligned} {\dot{r}}_1&= \frac{1}{2}[\gamma r_1 - 3 \beta \omega ^2 r_1^3 \nonumber \\&\quad + (r_1 - r_2 \cos \phi )(a + g(b,q) \, (r_1^2 + r_2^2 \nonumber \\&\quad - 2 r_1 r_2 \cos \phi ))], \nonumber \\ {\dot{r}}_2&= \frac{1}{2}[\gamma r_2 - 3 \beta \omega ^2 r_2^3 \nonumber \\&\quad + (r_2 - r_1 \cos \phi )(a + g(b,q) \, (r_1^2 + r_2^2 \nonumber \\&\quad - 2 r_1 r_2 \cos \phi ))], \nonumber \\ r_1 r_2 {\dot{\phi }}&= \frac{1}{2}(r_1^2 + r_2^2) \sin \phi \bigl [a + g(b,q) (r_1^2 + r_2^2 \nonumber \\&\quad - 2 r_1 r_2 \cos \phi )\bigr ]. \end{aligned}$$

As with the general damping terms, any additional general coupling terms (76) are additive.

### E.3 The general class of HKB models

We now present the most general class of HKB model, for coupled oscillators with *n* general nonlinear damping terms and *m* general nonlinear coupling terms, of the form:

87 $$\begin{aligned} \ddot{x}_1 + \omega ^2 x_1&= \epsilon \left[ \left( \gamma - \sum _{i=1}^n \beta _i |x_1|^{2 - 2p_i}|{\dot{x}}_1|^{2p_i}\right) {\dot{x}}_1 \right. \nonumber \\&\quad + ({\dot{x}}_1 - {\dot{x}}_2)(a \nonumber \\&\quad \left. \left. + \sum _{i=1}^m b_i |x_1 - x_2|^{2 - 2q_i}|{\dot{x}}_1 - {\dot{x}}_2|^{2q_i}\right) \right] , \nonumber \\ \ddot{x}_2 + \omega ^2 x_2&= \epsilon \left[ \left( \gamma - \sum _{i=1}^n \beta _i |x_2|^{2 - 2p_i}|{\dot{x}}_2|^{2p_i}){\dot{x}}_2 \right. \right. \nonumber \\&\quad + ({\dot{x}}_2 - {\dot{x}}_1)(a \nonumber \\&\left. \left. \quad + \sum _{i=1}^m b_i |x_2 - x_1|^{2 - 2q_i}|{\dot{x}}_2 - {\dot{x}}_1|^{2q_i}\right) \right] \end{aligned}$$

Using two-timing Cass (2019), these equations can be written in terms of amplitude and phase variables $r_1, r_2, \phi$:

88 $$\begin{aligned} {\dot{r}}_1&= \frac{1}{2}\biggl [\left( \gamma - \sum _{i=1}^{n} g(\beta _i, p_i) r_1^2\right) r_1 \nonumber \\&\quad + (r_1 - r_2 \cos \phi )\bigl (a \nonumber \\&\quad + \sum _{i=1}^{m}g(b_i,q_i) \, (r_1^2 + r_2^2 - 2 r_1 r_2 \cos \phi )\bigr ) \biggr ], \nonumber \\ {\dot{r}}_2&= \frac{1}{2}\biggl [\left( \gamma - \sum _{i=1}^{n} g(\beta _i, p_i) r_2^2\right) r_2 \nonumber \\&\quad + (r_1 - r_2 \cos \phi )\bigl (a \nonumber \\&\quad + \sum _{i=1}^{m}g(b_i,q_i) \, (r_1^2 + r_2^2 - 2 r_1 r_2 \cos \phi )\bigr )\biggr ], \nonumber \\ r_1r_2{\dot{\phi }}&= \frac{1}{2}(r_1^2 + r_2^2) \sin \phi \biggl [a \nonumber \\&\quad + \sum _{i=1}^m g(b_i,q_i) \, (r_1^2 + r_2^2 - 2 r_1 r_2 \cos \phi )\biggr ]. \end{aligned}$$

We non-dimensionalise amplitudes $r_{1,2}$ and scale the time variable using

15 $$\begin{aligned} R_i = \frac{r_i}{r_I}, \, (i=1,2); \quad s=\frac{1}{2}\gamma t. \end{aligned}$$

where now

89 $$\begin{aligned} r_I&= \sqrt{\frac{\gamma }{\sum _{i=1}^n g(\beta _i,p_i)}}, \end{aligned}$$

is the amplitude of the limit cycle, to give

16 $$\begin{aligned} \dot{R_1}&= R_1 - R_1^3 + (R_1 - R_2 \cos \phi )(\mu \\&\quad + \kappa (R_1^2 + R_2^2 - 2 R_1 R_2 \cos \phi )) \\ \dot{R_2}&= R_2 - R_2^3 + (R_2 - R_1 \cos \phi )(\mu \\&\quad + \kappa (R_1^2 + R_2^2 - 2 R_1 R_2 \cos \phi )) \\ R_1R_2{\dot{\phi }}&= \left( R_1^2 + R_2^2\right) \sin \phi \bigl [\mu \\&\quad + \kappa (R_1^2 + R_2^2 - 2 R_1 R_2 \cos \phi )\bigr ] \end{aligned}$$

where $\mu =\frac{a}{\gamma }$ as before, (17), and $\kappa$ is now given by

90 $$\begin{aligned} \kappa&= \frac{d}{\delta }=\frac{\sum _{i=1}^m g(b_i, q_i)}{\sum _{i=1}^n g(\beta _i, p_i)}, \end{aligned}$$

where we now define the combined nonlinear coupling coefficient *d* as

91 $$\begin{aligned} d&= \sum _{i=1}^m g(b_i, q_i) \end{aligned}$$

and the combined nonlinear damping coefficient $\delta$ as

92 $$\begin{aligned} \delta&= \sum _{i=1}^n g(\beta _i, p_i), \end{aligned}$$

When $n=2, \, \beta _1=\alpha , \, p_1=0, \, \beta _2 = \beta , \, p_2 = 1$ and $m=2, \, b_1=b, \, q_1=0, \, b_2=c, \, q_2=1$, (87) reduces to (10). Also (89), (90) reduce to (7), (18), respectively, and (91), (92) reduce to (19), (20), respectively.

Hence, we conclude that a very large class of biologically relevant weakly damped, weakly coupled HKB models (87) can be represented by one set of nondimensionalised equations (16) involving only two dimensionless parameters $\mu$, defined in (17), and $\kappa$, defined in (90).

## F Dimensional quantities

In Table 2, we gave analytic expressions derived from the approximate aHKB model (16) for $I, \, A, \, L^{\pm }$, $\, N^{\pm }_0, \, N^{\pm }_{\pi }$ in terms of the dimensionless parameters $\mu , \, \kappa$. The *dimensional* versions of these quantities that we use in our comparison with our numerical results are given by:

93 $$\begin{aligned} {\hat{I}}&= 2\sqrt{\frac{\gamma }{13}}, \end{aligned}$$

94 $$\begin{aligned} {\hat{A}}&= 2\sqrt{\frac{\gamma +2a}{13-8b}}, \end{aligned}$$

95 $$\begin{aligned} {\hat{L}}^{\pm }&= {\hat{I}} = 2\sqrt{\frac{\gamma }{13}}, \end{aligned}$$

96 $$\begin{aligned} {\hat{N}}^{\pm }_0&= \frac{1}{\sqrt{13(b+13)}}\left[ \sqrt{13\gamma +39a+4b\gamma }\pm \sqrt{13(\gamma -a)}\right] , \end{aligned}$$

97 $$\begin{aligned} {\hat{N}}^{\pm }_{\pi }&= \frac{1}{\sqrt{13(b+13)}}\left[ \sqrt{13(\gamma -a)} \pm \sqrt{13\gamma +39a+4b\gamma }\right] . \end{aligned}$$

Note that (93), (94) is (7), (9) evaluated at $\alpha =\beta =1, \, \omega =2$ and (95) is the dimensional version of (31).

## Footnotes

1 Sometimes referred to as the slowly varying amplitude and rotating wave approximations.

2 By convention damping, coefficients are positive when energy is removed from the system.

3 The factor of $\frac{1}{4}$ in both *d* and $\delta$ is convenient when we consider the general HKB model in Appendix E.

4 Other scalings are possible. The choice (15)–(18) is quite natural since the damping coefficients $\gamma , \, \alpha , \, \beta$ are intrinsic to the oscillators. In an experiment, it is hard to imagine that the linear damping coefficient $\gamma =0$, ensuring that $\mu$ would always be finite. On the other hand, the coupling coefficients $a, \, b, \, c$ are more likely to change, to include the uncoupled case $a=b=c=0$, corresponding to $\mu =\kappa =0$.

5 Table 2, rows 1–5 correspond to (Leise and Cohen 2007, Table 1, rows 2–6). Table 2, rows 7–9 cover (Leise and Cohen 2007, Table 1, row 1). But (Leise and Cohen 2007, Table 1) has no equivalent of Table 2, row 6. It can be shown that (Leise and Cohen 2007, Table 1, row 7) is not a solution of (16).

6 AUTO is a widely used computer package that detects bifurcations and other nonlinear phenomena, by treating a set of parameterized ordinary differential equations and their initial conditions as a boundary value problem. There are currently more than a dozen similar packages available.

7 In Avitabile et al. (2016), it is stated that $\omega =0.2$. However, we reproduced their results by setting $\omega =2$. Other parameter values were also used in that paper.

## Declarations

### Code availability

(software application or custom code): Not applicable
